# What Value Do Dutch Citizens Place on Health Interventions That Provide Greater Health Gains to Lower-Income Groups? A Discrete Choice Experiment

**DOI:** 10.34172/ijhpm.9095

**Published:** 2026-02-15

**Authors:** Iris Meulman, Adrienne Rotteveel, Ellen Uiters, Mariëlle Cloin, Johan Polder, Niek Stadhouders

**Affiliations:** ^1^Center for Public Health, Healthcare and Society, National Institute for Public Health and the Environment, Bilthoven, The Netherlands.; ^2^Tranzo, Tilburg School of Social and Behavioral Sciences, Tilburg University, Tilburg, The Netherlands.; ^3^Netherlands School of Public & Occupational Health, Utrecht, The Netherlands.; ^4^Scientific Center for Quality of Healthcare, Radboud University Medical Center, Nijmegen, The Netherlands.; ^5^Department of Health Economics, School of Business and Economics & Talma Institute, Vrije Universiteit, Amsterdam, The Netherlands.

**Keywords:** Discrete Choice Experiment, Healthcare Resource Allocation, Socioeconomic Health Inequality, Equity-Efficiency Trade-Off, Prevention, Priority Setting

## Abstract

**Background:**

Reimbursement decisions for new health interventions focus on maximizing health gains, with limited attention to who benefits from these gains or the impact on income related health inequalities. This study aimed to examine the preferences of Dutch citizens regarding the distribution of health gains of new interventions across income groups.

**Methods:**

A discrete choice experiment (DCE) was completed by 614 Dutch adults. Respondents were presented with 12 choice tasks. In each choice task, they were asked to choose between two health interventions that differed on the following attributes: total healthy life years gained, distribution of healthy life years gained across income groups, additional costs in terms of health insurance premium increases and whether the intervention was curative or preventive. Preferences were estimated using multinomial logit (MNL) models, relative attribute importance, willingness-to-pay, and willingness-to-trade total health gains. Preference heterogeneity was examined using latent class (LC) analyses.

**Results:**

Respondents found the distribution of health gains by income the most important attribute in their decision between health interventions (relative importance [RI] = 40.5%, 95% CI: 38.3%–42.7%). Overall, respondents preferred an equal distribution of healthy life years gained across income groups (β_higher income groups_ = -1.427, 95% CI: -1.547–-1.307; β_lower-income groups_ = -0.315, 95% CI: -0.395–-0.235). A health intervention should yield 14 283 (95% CI: 10 463–18, 102) additional healthy life years or reduce the yearly health insurance premium by €39.96 (95% CI: €29.03–€50.89) if it mainly favors lower-income groups. Preventive interventions were generally preferred over equally effective or more effective curative interventions (β_prevention_ = 0.270, 95% CI: 0.204–0.336). While preferences displayed a similar direction across LCs, the classes differed in the RI assigned to the attributes.

**Conclusion:**

Our findings suggest societal support for interventions that prioritize preventive programs over equally effective or more effective curative interventions and prioritize interventions that provide equal benefits across different income groups.

## Background

**Key Messages**

**Implications for policy makers**

- With health resources being limited, understanding how citizens prioritize different aspects of health interventions can help policy-makers to better align allocation of resources with the values and priorities of the population.
- Our findings indicate that policies disproportionately benefitting higher-income groups may not align with the preferences of the Dutch population, which can be used by policy-makers to inform decision-making.
- If the government aims to align policy-making with general societal preferences, our findings suggest to prioritize preventive interventions over equally or more effective curative interventions.
- While societal views and preferences for health intervention reimbursement were found to be largely shared, different groups may prioritize different aspects. If policy-makers want to develop healthcare allocation and reimbursement policies that are responsive to different societal priorities and broadly supported, they can use insights from our study to inform their decision-making.

**Implications for the public**

Due to limited financial and human resources, choices in the reimbursement of prevention and care are inevitable. In this study, we asked Dutch citizens which aspects of health interventions (total health gains, distribution of health gains between income groups, costs and type of intervention) they considered important for healthcare allocation decisions made by the government. The results indicated that there is societal support for a focus on prevention and for ensuring that health gains are distributed equally between income groups. Most preferences were widely shared among respondents, yet some groups placed greater importance to specific aspects of health interventions than others. These valuable insights into public preferences may inform health policy decision-making.

Like in many other countries, the healthcare system in the Netherlands is pressured by limited financial and human resources, challenging its principles of affordability and solidarity. Existing constraints on healthcare spending necessitate prioritization of health interventions covered by mandatory health insurance.^1^ The benefits of interventions are often measured in quality-adjusted life years (QALYs), which quantify health gains in terms of both extended lifespan and improved quality of life.^2^ In the Netherlands, interventions are reimbursed that meet the criteria effectiveness, cost-effectiveness, necessity and feasibility.^3,4^ The reference value to determine if interventions are cost-effective ranges from €20 000 per QALY for treatments targeting patients with a low disease burden and €80 000 per QALY for those addressing a high disease burden.^5-7^ Recently, an uniform reference value of €50 000 per QALY was recommended for preventive interventions.^8,9^ The current reimbursement decision-making process does not explicitly consider the distribution of health gains across different subgroups within the population. This is partly because principles such as justice and solidarity are ethically important but difficult to operationalize in a standardized way within the decision-making framework. The process emphasizes overall health gains and cost-effectiveness at the population level, even though this may sometimes disadvantage specific subgroups. Addressing such trade-offs is seen as requiring broader societal debate rather than technical criteria alone.^10^

 Considering the distribution of health gains across subgroups may be particularly relevant given the large differences in (healthy) life expectancy between income groups. On average, individuals within the 20% highest income category live 7.7 years longer and 21.4 years longer in good health than those in the 20% lowest income class.^11^ A general public aversion to income related health inequalities exists.^12-15^ Addressing these inequalities in health, however, may require policies disproportionally benefitting lower-income groups.^16-21^ In this paper we therefore aim to examine the differences in societal support for healthcare allocation with varying health gains across income groups.

 Diverse citizens’ views on healthcare allocation across groups have been revealed by several Dutch studies. One study on deservingness found a preference for allocating more resources to lower-income groups.^22^ However, a Dutch citizens’ forum showed people are willing to set healthcare reimbursement criteria based on condition, treatment, and personal characteristics^23^ without explicitly referencing to income or socioeconomic status. More broadly, van Exel et al^24^ identified five viewpoints on healthcare priority setting, of which the viewpoint “egalitarianism, entitlement, and equality of access” was supported by 44% of the population.^24,25^

 In the UK, studies highlighted the aversion to health inequalities, and especially those tied to non-health related characteristics. McNamara et al^26^ demonstrated that UK citizens are more willing to prioritize health improvements for deprived socioeconomic groups compared to neutrally labelled groups. Similarly, Robson et al^27^ demonstrated a stronger aversion to income related health inequality than to pure health inequality. When comparing other non-health characteristics, there was a weak preference for prioritizing health of females, moderate for individuals with lower income, and strong for non-smokers over smokers. These findings have been attributed to the perceived level of individual responsibility for income and smoking status.^28^ Contrarily, a Canadian study showed that respondents preferred general population interventions over those tailored to marginalized communities.^29^

 Societal preferences for distributing (healthy) life year gains across income groups can be measured by what people are willing to sacrifice to realize these preferences. One approach is the equity-efficiency trade-off, weighting equality in resources or health outcomes against total health gains. A recent US review found modest support for equal resource distribution and sacrificing total health gains for equity based on four studies: 26%-78% favored equal distribution, and 8%-25% prioritized disadvantaged groups.^30^ However, Blacksher et al^31^ reported that US participants moderated their distributive preferences to maximize health and prioritize the sickest regardless of their socioeconomic status. Distributive preferences can also be assessed by willingness to pay (WTP) or willingness to increase health insurance premiums. Gongora-Salazar et al^32^ found in England a positive WTP for treating populations with more low-income individuals. Boujaoude et al^33^ showed that Australian respondents valued incremental health gains to the poorest fifth five times more than to the richest fifth.

 In addition to preferences for the distribution of health gains, individuals may have distinct preferences for the types of health interventions used to achieve health gains. Literature is inconclusive on individuals’ preferences for preventive care (like vaccinations and screenings) and curative care (treatments).^34-36^ Wolff et al^37^ found that among a sample of the Swedish general population, more people were unwilling to pay extra for prevention compared to curative treatment, although average WTP for prevention was 85% higher. A recent Dutch study showed that respondents had greater preference for increasing healthcare expenditure for curative care (especially elderly care, new and better medicines and mental healthcare) than for preventive care.^38^ However, a Dutch study from the early 2000s indicated no difference in value for prevention, cure, and care.^39^ A Canadian discrete choice experiment (DCE) found that respondents were less likely to choose preventive programs compared to treatment.^29^

 With health resources being limited, understanding how citizens prioritize different aspects of health interventions can help policy-makers to better align allocation of resources with the values and priorities of the population. The diversity of preferences and perspectives on healthcare allocation and reimbursement brought forward by the aforementioned studies suggests that these preferences may not be uniform among the population. This study expands upon previous literature by combining the equity-efficiency trade-off with a health-income trade-off and treatment type, using a large sample of representative respondents in a relative equitable health system. The aim of this study was to reveal the preferences of Dutch citizens for (1) the total gains in healthy life years of health interventions, (2) the distribution of health gains of health intervention by income groups, (3) increase in health insurance premium, and (4) the type of health intervention. The following sub-questions were formulated:

1) Which preferences do Dutch citizens have regarding the total health gains, distribution of health gains across income groups, health insurance premium, and type of intervention? And how do they trade-off these attributes?
  - Are Dutch citizens willing to pay more or reduce total gains in healthy life years if a larger proportion of the health gains accrues to lower-income groups?
  - Do Dutch citizens value preventive health interventions differently than curative health interventions?
2) To what degree do preferences for health interventions differ across groups of individuals?

## Methods

 We designed a questionnaire including a DCE to examine the preferences of Dutch citizens towards the relative importance (RI) characteristics and cost of health interventions. By “health interventions,” we mean a wide range of possible actions, treatments, programs, policies, or health services that can be both curative and preventive. In a DCE, respondents are presented with choice tasks and repeatedly choose between alternative options (eg, policies, health interventions, products) defined by specific attributes. Responses to these choices reveal the value of both the options and their attributes. DCE’s have commonly been used in health economics to examine public and patient preferences and are particularly suitable for examining the preferences of citizens regarding various health intervention attributes because they effectively capture trade-offs between multiple attributes, provide quantitative and policy-relevant insights, and identify preference heterogeneity.^40^

###  Data Collection 

 For the data collection, we made use of the LISS (Longitudinal Internet studies for the Social Sciences) panel administered by Centerdata (Tilburg University, The Netherlands), which covers approximately 7500 individuals, representative of the Dutch general public, aged 18 years and older. The LISS-panel is derived from a random sample of the Dutch population registry, without the possibility of self-registration. Random or stratified additions are made to compensate for dropouts. All respondents received €3.00 compensation for filling in the questionnaire. Data collection for the main questionnaire took place in July 2024. The research project has been assessed by the Centre for Clinical Expertise (CCE) at the National Institute for Public Health and the Environment (RIVM) in the Netherlands. The CCE concluded that the research project is exempted from further review by a medical ethics committee as it does not fulfil the specific conditions as stated in the Dutch Medical Research Involving Human Subjects Act.

###  Questionnaire

 The questionnaire consisted of four parts. First, respondents were introduced to the topic. The second part consisted of questions regarding work situation, difficulties making ends meet and self-rated health. Other descriptive characteristics of the sample were obtained from the panel organization. The third section comprised the DCE, which included an explanation of the choice tasks, attributes, and levels, as well as the choice tasks. This section ended with follow-up questions on the choice tasks, including ranking the importance of attributes and two open-ended questions asking participants why they considered this attribute most important and whether they considered other attributes when choosing between the health interventions. Fourth, the questionnaire concluded with 19 evaluative statements and an open question for comments about the questionnaire in general. The full questionnaire is presented in Supplementary file 1.

###  Design of the Discrete Choice Experiment 

 In the DCE, respondents were asked to choose between two health interventions, each described with attributes and levels. These interventions could each provide benefits for 10 000 patients. This group of 10 000 patients was then divided into two equal sized groups based on their income: the group with the 50% lowest incomes and the group with the 50% highest incomes. Therefore, there was no middle-income group. Respondents were presented with 12 choice tasks, each time deciding which of the two health interventions should be reimbursed as part of the basic benefits package. In the Netherlands, all persons have access to a statutory benefits package through mandatory health insurance.

**Figure 1:**
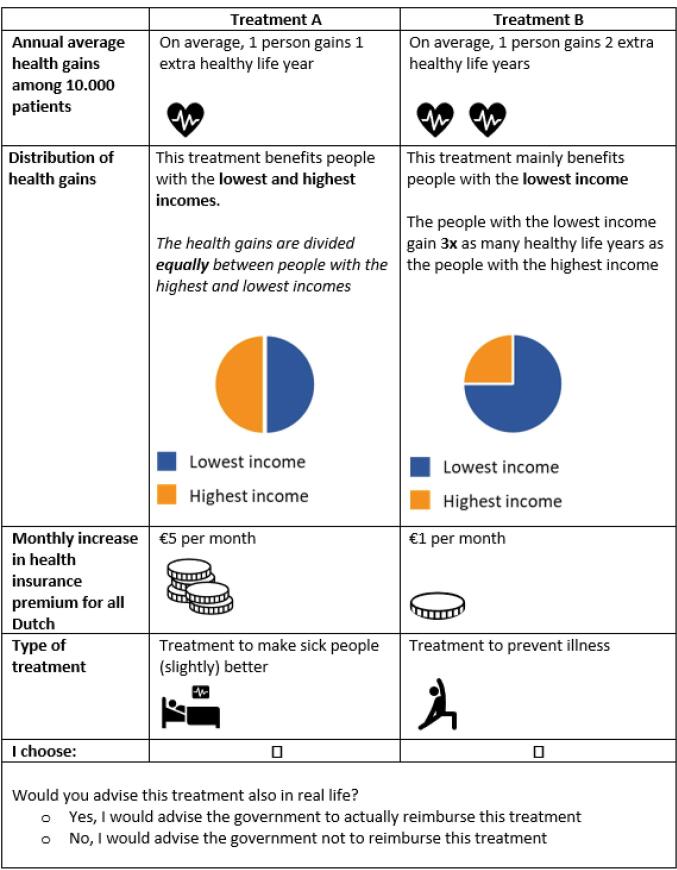
Example of a Discrete Choice Task.

 Potentially relevant attributes were identified through a literature search using terms like health inequality, equity, efficiency, health benefits, QALY, WTP, trade-off, DCE, cost-effectiveness analysis, decision-making, priority-setting, and preferences. Fifteen relevant articles were selected, yielding 23 characteristics of health interventions (Supplementary file 2). A first selection based on relevance to the research question, distinctiveness, and simplicity reduced this to 14 attributes. Finally, for each characteristic of the health interventions mentioned in the research question, one attribute was selected based on its relevance and synergistic compatibility. See Table 1 for the selected attributes and levels. Attribute levels were based on societal relevance, customary values and literature. The levels for “average health gains for 10 000 patients” and “health insurance premium increase” were designed to be comprehensible and practical for respondents, while covering a range of WTP per QALY-values including the cost-effectiveness thresholds used in the Netherlands. Average healthy life years gained were set at 1, 2, or 5 years, translating to 10 000, 20 000, or 50 000 healthy life years per year for 10 000 patients. The attribute “distribution of total health gains by income level” described how these total health gains were allocated among the 10 000 patients with different incomes, which could be equal, favoring higher incomes (distribution 25% for lower incomes and 75% for higher incomes), or favoring lower incomes (distribution 75% for lower incomes and 25% for higher incomes). The health insurance premium increase was presented as a monthly increase of €0, €1, €5, or €10 (the minimum monthly health insurance premium in the Netherlands was €140 in 2023^41^). The type of health intervention could be preventive or curative. Figure 1 provides an example of a choice task. Pictograms were used to improve clarity.

**Table 1:** Attributes and Levels of the Discrete Choice Tasks

**Attributes**	**Levels**
Annual average gain in healthy life years for 10 000 patients	1 healthy life year, 2 healthy life years, 5 healthy life years
Distribution of total health gains by income level	The health intervention equally benefits people with the lowest and highest income (distribution: 50%-50% of health gains) The health intervention mainly favors people with the highest income (25%-75%) The health intervention mainly favors people with the lowest income (75%-25%)
Increase in monthly healthcare premium	€0, €1, €5, €10
Type of health intervention	Curative, preventive

 Following the number of attributes and levels of Table 1, a complete factorial design would consist of 72 choice tasks (3*3*4*2) and 2556 sets of two choice tasks (72!/(2*(72-2)!)).^42^ Addressing all possible combinations of attribute levels and combinations of choice sets would be too costly and demanding for respondents. Therefore, a Bayesian D-efficient design was developed using the R-package idefix^43^ to reduce the number of choice tasks per respondent. Based on a predefined number of choice sets and prior parameter values, a Bayesian D-efficient with modified Fedorov optimalization algorithm design is used to generate and construct an optimal set of choice tasks. The objective of this approach is to maximize statistical efficiency of the design and minimize the required sample size.

 The order of alternatives, attributes and choice sets were randomized between respondents to minimize bias of ordering effects.^44^ One constraint was imposed whereby the health gains attribute was consistently followed by the distribution attribute, as we felt this would facilitate comprehension of the choice tasks for respondents. Choice tasks were unlabeled. To maximize obtaining information while allowing the option to decline reimbursement for both health interventions, a stepwise opt-out question (ie, dual-response) was asked after each choice set, yielding “Would you also choose this health intervention in real life?”

 Responses were classified as unreliable and excluded from analysis if both of the following criteria were met: (1) the respondent consistently selected either option A or B in at least 11 out of 12 choice tasks (ie, straight-lining), and (2) completed the survey within the fastest 10% of respondents (ie, speeders). Thus, we considered the response unreliable if classified as both straight-lining and speeding. Additionally, we analyzed responses to the open-ended survey evaluation question to further assess response reliability. For example, we considered indications of technical issues that may have influenced answers or self-reported concerns regarding the reliability of their own responses. Responses were excluded if we considered responses unreliable based on the open-ended response. Responses were classified as incomplete if participants did not complete the entire questionnaire.

###  Optimization of the Questionnaire 

 The preliminary questionnaire was pre-tested in face-to-face one-on-one sessions with participants and researchers to examine comprehension and feasibility on May 1, 2024. Participants were recruited by an external commercial panel organization (Norstat). In total, the questionnaire was pre-tested by 15 participants (sex, age, education representative). A combination of think-aloud and debriefing was used: participants were asked to complete the questionnaire on paper and express their thoughts and considerations out loud. After completing the questionnaire, debriefing questions were asked. Minor adjustments were made to the phrasing and length of the questionnaire following the pre-tests. Furthermore, the clarity of pictograms was discussed and participants were presented with three alternative visualizations of the attribute “distribution of health gains across income groups.” The visualization deemed most clear was subsequently employed in both the pilot and main study. Subsequently, Centerdata converted the questionnaire into an online format, and minor adjustments were made based on their recommendations. A pilot was conducted between June 1 and June 8, 2024, to optimize the questionnaire and calculate the minimum required sample size.^45^ The prior parameter values of the D-efficient Bayesian design were set to zero for the pilot study. After a pilot study, we changed the formulation of the health gains attribute to clarify that it was an annual average health gain for 10 000 patients. Furthermore, prior values were updated for the final questionnaire using the estimates obtained from a multinomial logit (MNL) model fitted on the responses of the pilot study, leading to 12 new choice sets. The minimum required sample size was 375 respondents.^45^ Pilot and main study data will be pooled if questionnaires were sufficiently comparable and the coefficient indicating data collection in the pilot or main study is statistically insignificant.

###  Statistical Analyses

 Discrete choice data was analyzed in the context of the random utility maximization framework, under the assumption that individuals choose the alternative that maximizes their expected utility per choice task.^46^ Costs and benefits were analyzed as continuous attributes, whereas the distribution of health gains by income and the type of health intervention were dummy coded. Costs were multiplied by 12 to reflect yearly increase in health insurance premium and align with yearly health gains. Main effects were derived using an MNL-model. The utility (U) of individual *i* choosing alternative *j* is given by:


(1) $$\begin{array}{l} U_{ij}=\beta_{0j}+\beta ’X_{ij}+\varepsilon_{ij}=\beta_{0j}+\beta_{1}\;Benefits_{ij}+\beta_{2}\;DistributionLI_{ij}+\\ \beta_{3}\;DistributionHI_{ij}+\beta_{4}\;Cost_{ij}+\beta_{5}\;Type_{ij}+\varepsilon_{ij} \end{array}$$

where β_0j_ reflects the alternative specific constants (ASCs), β’ the preference estimates for each attribute, X_ij_ the vector of attributes and ε_ij_ the error term accounting for unobserved factors and measurement error. To incorporate the dual-response option within the MNL-model, a forced-choice model and model with opt-out option are jointly estimated using a scaling parameter as described by Bradley et al.^47^ Following the MNL-model, the RI of attributes was calculated using the profile-based normalization approach (Equation 2).^48,49^


(2) $$RI_{X}=\frac{Max(PWU_{X})-Min(PWU_{X})}{\sum_{X}\left(Max(PWU_{X})-Min(PWU_{X})\right)}$$

where PWU_X_ refers to the part-worth-utility (the coefficient) of attribute X. Furthermore, the marginal WTP and marginal willingness to trade (WTT) total health gains was calculated by:


(3) $$Marginal\;WTP=-\frac{\beta_{X}}{\beta_{\cos t}}$$

(4) $$Marginal\;WTT\;total\;health\;gains=\frac{\beta_{X}}{\beta_{benefits}}$$

A latent class (LC) choice model was applied to identify groups of respondents with similar preferences. A LC choice model captures the heterogeneity of preferences across a number of unobserved classes c by a utility function with class-specific preference estimates (β-coefficients). The probability of a sequence of choice for individual i choosing alternative j in choice task t is given by:


(5) $$P_{i}\left(\beta_{1},\;...,\beta_{C}\right)=\sum_{c=1}^{C}\pi_{ic}\prod_{t=1}^{T_{i}}P_{j_{it}}\left(\beta_{c}\right)$$

where π_ic_ is the probability of individual i belonging to class c (indicating posterior class membership) and P_jit_ is the probability of individual i choosing alternative j in choice task t given class c. Starting values were determined using the starting value search algorithm in Apollo.^50^ LC choice models were estimated using the Broyden–Fletcher–Goldfarb–Shanno estimation algorithm for one to nine LCs. The optimal number of classes was determined by comparing Bayesian information criterion (BIC) and the distinctiveness and interpretability of preference profiles for the classes. BIC is a statistical measure to assess model performance by balancing accuracy and overfitting, where lower values indicated a better penalized model fit.^51^ Each individual was assigned to one of the identified classes based on their posterior class membership probabilities. The distribution of personal characteristics was, thereafter, examined across class membership. Marginal WTP and marginal WTT total health gains were calculated per class using equations 3 and 4.

 Statistical analyses were conducted using Apollo in R version 4.1.2.^52,53^ Robust standard errors and two-sided *P* values are computed using the sandwich estimator^50^ and 95% confidence intervals (CIs) of the RI, marginal WTP and marginal WTT total health gains were obtained using the delta-method.^54^

###  Analysis of Open-Ended Questions

 Participants answered open-ended questions about their highest-ranked attribute, other considerations in choosing between health interventions, and general comments on the questionnaire. Responses to these questions were analyzed by one researcher using thematic content analysis. Answers were inductively coded. Codes were then reviewed, merged or added as necessary. Summaries were created for each highest-ranked attribute argumentation, other considerations, and comments, focusing on rationales, aspects, and feedback deemed most important and frequently mentioned by participants. Answers to open-ended questions were both analyzed for the full sample, as well as for each LC separately. MAXQDA 24 was used for analysis of responses to open-ended questions.^55^

## Results

 The questionnaire was randomly distributed to 990 household members from the nationally representative LISS-panel. Of these, 137 completed the pilot questionnaire and 484 the main questionnaire, with a response rate of 68.1% and a completeness rate of 94.6%. Minimal adjustments after the pilot study and comparable estimates allowed pooling of pilot and main data (Supplementary file 3). Seven respondents were excluded due to straight-lining and speeding (n = 4), unreliability in the open-ended question (n = 2) or missing variables (n = 1), resulting in a final sample size of 614. The median completion time was 15.87 minutes.

 Men and women were equally represented which was reflective of the Dutch population aged 18 years and older (*P* < .9) (Table 2). The sample primarily comprised of respondents with high (43.32%) or middle (37.13%) educational levels, thereby overrepresenting higher educational attainment (*P* < .001). On average, respondents were 53.88 years old, being statistically older than the adult Dutch population (*P* < .001), and had a monthly net household income of €3995.21. Most respondents were employed, in good health, and had no difficulties making ends meet. Answers to the evaluation questions are presented in Supplementary file 4, for example showing that 67% of the respondents self-reported to have carefully read and included all information in their answers or that 57% of the respondents found the choices they had to make clear.

**Table 2:** Descriptive Statistics

**Characteristic**	**DCE Sample**^a^** (N = 614)**	**Dutch Population (N = 14 644 013)**^a,b^	***P*** ** Value**^c^
Sex			.9
Men	‎49.67% (305)‎	‎49.36% (7 227 ‎‎863)‎	
Women	‎50.33% (309)‎	‎50.64% (7 416 ‎‎150)‎	
Age (y)			<.001
18-24	‎5.70% (35)‎	‎11.02% (1 612 ‎‎854)‎	
25-34	‎13.84% (85)‎	‎16.23% (2 376 ‎‎706)‎	
35-44	‎15.47% (95)‎	‎15.09% (2 209 ‎‎693)‎	
45-54	‎11.40% (70)‎	‎15.68% (2 296 ‎‎084)‎	
55-64	‎18.73% (115)‎	‎16.85% (2 467 ‎‎032)‎	
>65	‎34.85% (214)‎	‎25.12% (3 677 ‎‎228)‎	
Mean age	‎53.88 (18.21)‎	‎49.67 (19.10)‎	<.001
Median age	‎57.00 (37.00; ‎‎69.00)‎	‎50.00 (33.00; ‎‎65.00)‎	<.001
Education^d^			<.001
Low	‎19.54% (120)‎	‎28.36% (4 200 ‎‎000)‎	
Middle	‎37.13% (228)‎	‎36.01% (5 332 ‎‎000)‎	
High	‎43.32% (266)‎	‎35.63% (5 277 ‎‎000)‎	
Mean household monthly net income	‎3995.21 (2169.65)‎		
Unknown (n)	‎63‎		
Median household monthly net income	‎3595.00 (2500; ‎‎5250)‎		
Unknown (n)	‎63‎		
Household monthly net income			
<€2000	‎15.79% (87)‎		
€2000-3000	‎21.42% (118)‎		
€3000-4000	‎19.24% (106)‎		
€4000-5000	‎14.34% (79)‎		
€5000-6000	‎13.25% (73)‎		
>€6000	‎15.97% (88)‎		
Unknown	‎63‎		
Difficulties making ends meet			
No difficulties	‎58.31% (358)‎		
No difficulties, but minding expenses	‎30.46% (187)‎		
Some/large difficulties	‎11.24% (69)‎		
Self-reported health			
(Very) good	‎68.89% (423)‎		
Average health	‎27.52% (169)‎		
(Very) bad	‎3.58% (22)‎		
Employment situation			
Employed	‎49.19% (302)‎		
Retired	‎33.39% (205)‎		
Unemployed	‎5.86% (36)‎		
Study	‎11.56% (71)‎		
Median completion time (min)	‎15.87 (10.84; ‎‎28.21)‎		

Abbreviation: DCE, discrete choice experiment. ^a^ % (n); mean (SD); median (25%; 75%). ^b^ Dutch population at January 1, 2024. Data source: CBS StatLine. ^c^ Pearson’s chi-squared test. ^d^ Distribution of education in Dutch population based on numbers in 2022 rounded by 1000.

###  Utility and Relative Importance of Attributes 

 The opt-out option was chosen in 23% of the choice tasks. 177 of the 614 respondents always chose the option to reimburse the intervention in real life (29%), while 8 respondents always opted-out (1%) (results not shown). The ASC for the opt-out option was statistically significantly negative, indicating that respondents derive negative utility (ie, dislike) from choosing the opt-out option rather than one of the alternative choices of reimbursing health interventions.

**Figure 2:**
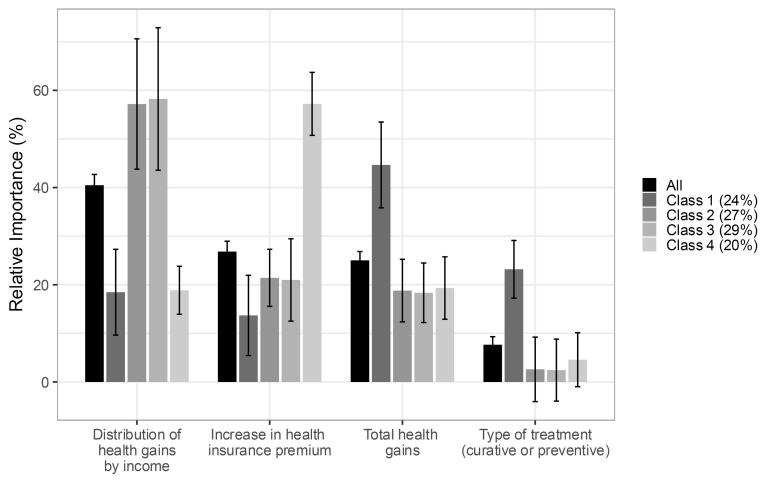
Relative Importance of Attributes for All Respondents and Per Latent Class. Class 1: “preferring lower-income groups benefitting more,” Class 2: “equal distribution of health gains,” Class 3: “maximizing health gains and prevention,” and Class 4: “cost-conscious.” The error bars indicate 95%-CIs.

 Respondents experienced lower marginal utility if higher or lower-income groups received greater health gains (β = -1.427, 95% CI: -1.547–-1.307 and β = -0.315, 95% CI: -0.395–-0.235, respectively) compared to an equal distribution. This means that respondents were more likely to choose a health intervention for reimbursement if the health gains are equally distributed than when higher or lower-income groups receive greater health gains. Also, utility was lower if health insurance premiums increased (β = -0.008, 95% CI: -0.009–-0.007) (Table 3 – MNL). Respondents obtained a positive marginal utility if the health intervention implied higher health gains (β = 0.221, 95% CI: 0.198–0.243) or was preventive instead of curative (β = 0.270, 95% CI: 0.204–0.336). The distribution of health gains by income was found the most important attribute in decision-making (RI = 40.5, 95% CI: 38.3–42.7), followed by an increase in health insurance premiums (RI = 26.8, 95% CI: 24.7–29.0), total health gains (RI = 25.0, 95% CI: 23.2–26.8) and type of health intervention (RI = 7.7, 95% CI: 6.0-9.3) (Figure 2 – All).

**Table 3:** Jointly Estimated Multinomial Logit Model

	**MNL**
	**β Coefficient (95% CI)**
**Attributes and covariates**	
Health gains (10 000 healthy life years)	0.221 (0.198-0.243)
Distribution of health gains by income	
Equally distributed (50/50)	Ref
Greater health gains for higher income groups (75/25)	-1.427 (-1.547–-1.307)
Greater health gains for lower income groups (25/75)	-0.315 (-0.395–-0.235)
Health insurance premium (€, yearly)	-0.008 (-0.009–-0.007)
Type of health intervention	
Curative	Ref
Preventive	0.270 (0.204-0.336)
ASC (alternative B)	0.041 (-0.006-0.088)
ASC (opt-out)	-0.572 (-0.695–-0.449)
Scaling parameter opt-out model	1.105 (1.059-1.151)
Scaling parameter pilot	1.097 (0.954-1.241)
**Model output**	
Number of observations	614
Final LL forced model	-4368.63
Final LL opt-out model	-7041.79
Final LL joint model	-11 410.42
AIC	22 838.84
BIC	22 900.99
Rho-squared	0.14

Abbreviations: MNL, multinomial logit; CI, confidence interval; ASC, alternative specific constant; LL, log-likelihood; AIC, Akaike information criterion; BIC, Bayesian information criterion.

###  Willingness to Pay and Willingness to Trade Total Health Gains

**Figure 3:**
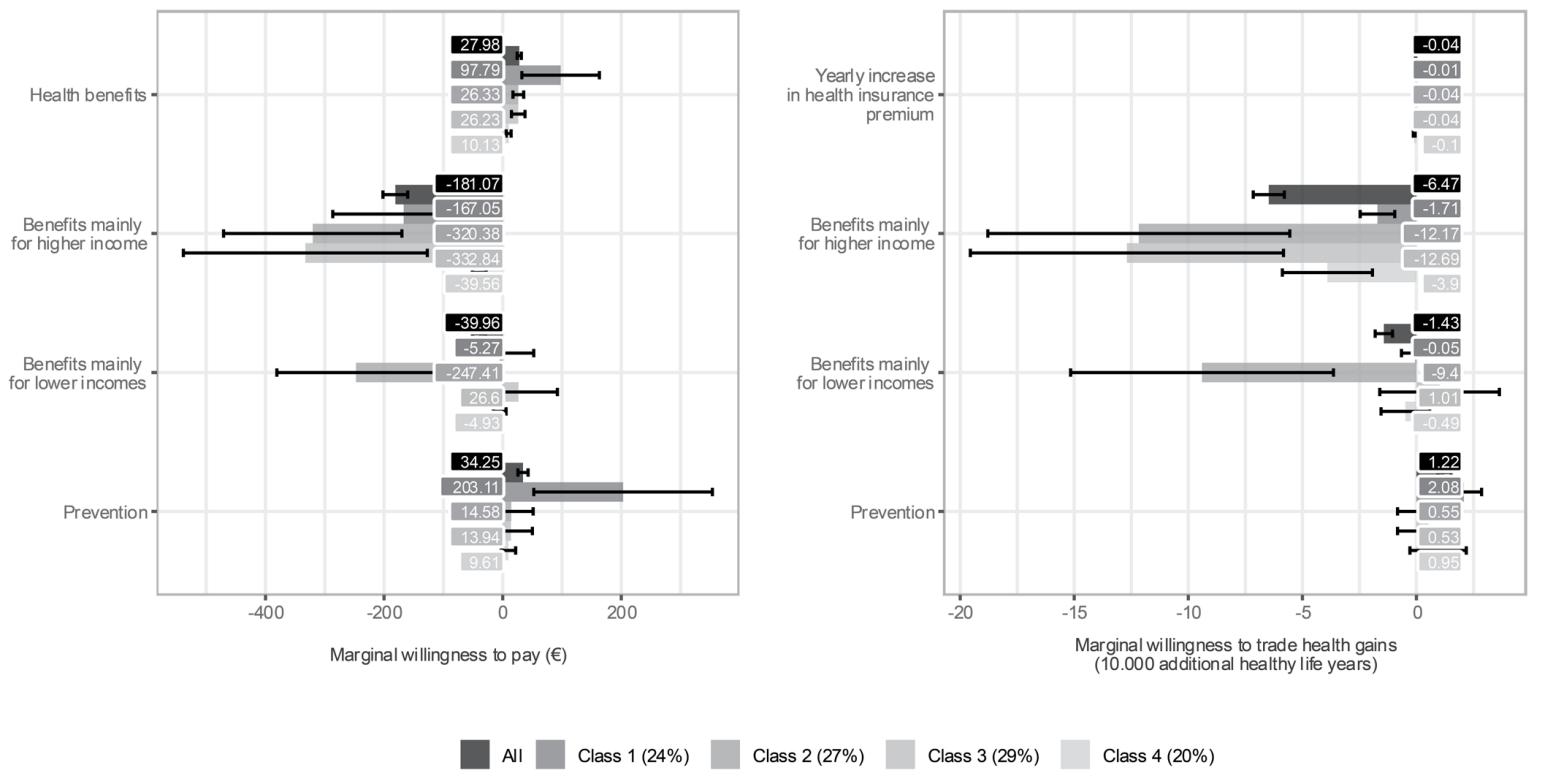
Marginal Willingness to Pay and Willingness to Trade Health Gains for All Respondents and Per Latent Class. Class 1: “maximizing health gains and prevention,” Class 2: “equal distribution of health gains,” Class 3: “preferring equal health gains or lower-income groups benefitting more,” and Class 4: “cost-conscious.” The error bars indicate 95%-CIs. Benefits mainly for higher income is compared to equally distributed health gains. Benefits mainly for lower income is compared to equally distributed health gains. Prevention is compared to curative health interventions.

 Deduced from the choice tasks, respondents wanted to receive 64,721 (95% CI: 57 901–71 541) additional healthy life years or a premium reduction of €1181.07 (95% CI: €160.15–€202.00) per year if the intervention mainly favored higher income groups compared to an equal distribution. Furthermore, a health intervention should yield 14 283 (95% CI: 10 463–18 102) additional healthy life years or reduce the premium by €39.96 (95% CI: €29.03–€50.89) per year if it mainly favors lower-income groups. Note that the marginal WTT total health gains for higher benefits for higher incomes is an out-of-sample extrapolation and may lack robustness. Additionally, respondents were willing to pay €34.25 (95% CI: €25.65–€42.84) more per year or forgo 12 241 (95% CI: 9323–15 159) healthy life years for preventive over curative interventions. Overall, respondents were willing to increase the health insurance premium by €27.98 (95% CI: €24.48–€31.48) per year per payer for an average gain of 1 healthy life year for 10 000 people (Figure 3 – All), equaling 10 000 healthy life years gained. Given 14.6 million health insurance premium payers in the Netherlands (Table 2), this indicated a societal WTP of approximately €41 000 per QALY.

###  Responses to Open-Ended Questions

 Respondents provided diverse reasons for prioritizing specific attributes in the study. Supplementary file 4 provides the code tree and more extensive findings of the qualitative analysis of questionnaire responses per attribute. The health gains attribute was valued for its fundamental importance to healthcare and individual well-being. Opinions on the distribution of health gains between income groups varied significantly. Some favored allocating greater health gains to lower-income individuals to reduce income related health inequalities, arguing that higher-income individuals can afford more on their own. Others supported equal healthcare rights and health chances irrespective of income or opposed giving greater health gains to higher incomes due to their already better position. Concerns about the high cost of premiums and their potential unaffordability for low-income individuals were also prominent. Several respondents traded premium increases against health gains. Preventive measures were often preferred, with arguments that prevention is better than cure, yields health benefits, and saves long-term costs. Curative care was valued for ensuring treatment when needed. Some participants highlighted that inefficiencies in healthcare, such as waste and misallocation of funds, should be addressed before deciding between health interventions. Furthermore, some expressed a desire to consider factors not included in the study, such as side effects, specific interventions, diseases targeted, and patient characteristics.

###  Latent Class Model 

 We estimated LC choice models to explore preference heterogeneity. Given the consistent decrease in BIC for 1-9 LC models without plateauing^51^ (Supplementary file 5), we selected a four-class model based on class distinctness and interpretability. Overall, the direction across classes was similar but with heterogeneity in attribute importance. For the distribution of health gains across income groups, the direction in preference across classes varied.

 Individuals most likely belonging to Class 1 (24% of respondents) strongly preferred maximizing total health gains and favored preventive over curative interventions. They showed positive marginal utility for preventive interventions and were willing to pay €203.11 (95% CI: €52.46–€353.76) more per year for them (Supplementary file 5 and Figure 3). These individuals found the total health gains attribute most important (RI = 45%, 95% CI: 36%–53%) and attached the most weight to the type of health intervention (RI = 23%, 95% CI: 17%-29%) of all classes (Figure 2). These observations were confirmed by the answers to the open-ended questions, although a few people in this group considered cure more important than prevention. This class, termed “maximizing health gains and prevention,” comprised of individuals who were more likely to be men, aged between 25 to 44 years and less likely to be retired or unemployed (Supplementary file 5).

 Individuals most likely belonging to Class 2 (27% of respondents) preferred an equal distribution of health gains, showing strong aversion to allocating greater health gains to either higher (β = -2.191, 95% CI: -2.725–-1.658) or lower-income groups (β = -1.692, 95% CI: -2.197–-1.187) and negative marginal WTP, and WTT total health gains (Supplementary file 5 and Figure 3). Respondents in this class quite unanimously mentioned “equality” as justification for their choices, leading to the label “equal distribution of health gains.” This class found the distribution of health gains among income groups most important attribute (57%, 95% CI: 44%–71%). Individuals most likely belonging to this class were more likely to be female and aged 18-24 years old (ref: >65 years) and chose the opt-out option most often (Supplementary file 5).

 Individuals most likely belonging to Class 3 (29% of respondents) preferred allocating equally or greater health gains to lower-income groups, showing statistically insignificantly positive values for marginal utility, WTP, and WTT total health gains for allocating greater health gains towards lower income, and statistically significant negative values for allocating greater health gains towards higher incomes (Supplementary file 5 and Figure 3). They, together with individuals most likely belonging to Class 4, chose the opt-out option least often and derived statistically significantly negative marginal utility from choosing the opt-out option. Their open-ended question responses indicated a focus on the vulnerable position of low-income individuals and a preference for healthcare policy to address this in some way, mentioning for example their health disadvantage, the affordability of premiums or the added value of prevention for this group. This class was termed “preferring equal health gains or lower-income groups benefitting more.” Individuals who were older, lower educated, had to mind expenses and are retired were more likely to belong to this class, while individual in (very) good health and living in households earning more than €6000 per month were less likely to belong to this class (Supplementary file 5).

 Individuals most likely belonging to Class 4 (20% of respondents) showed the strongest aversion towards increasing health insurance premiums (β = -0.027, 95% CI: -0.033–-0.022), resulting in low WTP for health benefits and prevention (Supplementary file 5 and Figure 3). They, together with individuals most likely belonging to Class 3, chose the opt-out option least often and derived statistically significantly negative marginal utility from choosing the opt-out option. Responses to the open-ended questions revealed concerns about the high cost and affordability of premiums. Individuals belonging to this “cost-conscious” class were less likely to lower educated, living in households earning €2000-3000 or €4000-5000, and be retired (Supplementary file 5).

## Discussion

 This study explored the preferences of Dutch citizens regarding health intervention characteristics using a DCE with 614 respondents. This sample was representative of the Dutch population in terms of sex distribution but was older and higher educated. Specifically, the study examined trade-offs between total health gains, distribution of health gains by income groups, increases in health insurance premiums, and the type of health intervention. Most respondents considered the distribution of health gains across income groups the most important factor, preferring equal distribution with respect to income. Overall, they were unwilling to pay higher premiums or sacrifice total health for a larger proportion of health gains accruing to either lower or higher income groups. Preventive care was valued over curative care.

 Four LCs with distinct preferences were identified: “maximizing health gains and prevention,” “equal distribution of health gains,” “preferring equal health gains or lower-income groups benefitting more,” and “cost-conscious.” In general, the direction of preferences was fairly consistent across the LCs, indicating a largely shared view on the allocation of scarce healthcare resources and reimbursement of health interventions. However, the different classes assigned varying weights to health intervention characteristics. The distribution of respondents across these classes was approximately equal, indicating that there was no single dominant preference pattern among Dutch citizens. These preferences showed some association with background characteristics like age, sex, income, education, or health status. Thus, while societal views and preferences were largely shared, different groups may prioritize different aspects. Policy-makers can use our findings to inform targeted information provision, public engagement (eg, citizen fora), and inclusion of patient perspectives in policy and guideline development. While differences in priorities may not always warrant separate policies, these approaches can help ensure broadly supported and responsive healthcare allocation and reimbursement decisions.

###  Insights into the Preferences Regarding the Distribution of Health Gains

 The general finding that Dutch citizens prefer an equal distribution of health gains means that respondents did not want to prioritize patients on the basis of income. These results are consistent with previous findings where most respondents disapproved of prioritizing low-income groups over others with the same health conditions.^24^ However, they contrast with other studies that found a willingness to prioritize lower-income groups^22^ or a WTP when a larger proportion of the target population consists of individuals with lower incomes.^32^ In our sample, this perspective was mentioned by a minority of respondents in the open-answer responses, primarily those in the “preferring lower-income groups benefiting more” class.

 The debate surrounding equality and equity in healthcare, such as the equity-efficiency trade-off, is complicated by varying interpretations, different operationalizations, and the interchangeable use of terms.^30,56^ Commonly discussed concepts include equal treatment for equal need, equality of access, opportunity or resources, equality of health outcomes, and prioritization of the worse-off. In our study, we approached this by examining the distribution of health gains across income groups. Pursuing an equal distribution of health gains may have implications for the distribution of resources, opportunities and health outcomes,^57^ which we will discuss in the following paragraphs. However, these implications are not directly measured in our study. Furthermore, the discussion surrounding the distribution of health gains across income groups assumes that the effectiveness of interventions for different groups is well understood and can be adjusted accordingly. However, this may be unlikely, as effectiveness (across groups) may be difficult to prove, be influenced by contextual factors and only become apparent over the long term.^18,58,59^

 Despite this, our findings indicated that Dutch citizens were willing to forgo maximizing health gains if it conflicted with their preferences for the distribution of health gains. To better understand the dynamics of the equity-efficiency debate, one should compare these findings with the results when repeating the experiment but using instead other operationalizations such as the distribution of health outcomes across income groups or distribution of opportunity or resources. Further insights could also be gained by considering the different healthcare needs of the patients who received the intervention.

 Our findings indicated that respondents across all LCs strongly disapproved of health interventions that disproportionately benefit higher-income individuals. Responses to open-ended questions revealed that respondents felt high-income groups already have more resources for a healthy lifestyle and better access to healthcare, and therefore should not receive additional benefits over others. However, some universal health policies, despite allocating resources equally, may unintentionally favor higher-income groups. This may be because higher-income individuals are often better equipped to engage with or adopt new interventions and utilize resources more effectively.^16-21^ Since policies that disproportionately benefit higher-income groups do not align with the preferences of the Dutch population, policy-makers are advised to ensure equal health gains across all income groups. Achieving this may require allocating disproportionately more resources to individuals with lower incomes to obtain equal health gains across income groups. However, further research is needed to directly confirm whether allocating resources disproportionately to achieve equal health gains aligns with societal preferences.

 With regard to health outcomes, the difference in healthy life year expectancy between income groups is currently 20 years and has not changed much over the last years.^11,60^ The general preference of respondents for distributing healthy life years gained from health interventions equally between income groups would imply that the absolute gap in healthy life years between these groups will remain unchanged. However, given the lower overall healthy life expectancy of lower-income groups, achieving equal additional healthy life years for both groups would reduce relative inequality (a reversed mathematical artifact as described by Mackenbach^19^). By ensuring that all income groups receive the same amount of health improvement, the proportional difference in healthy life years between higher and lower-income groups will be reduced. Further research is needed to more directly measure the preferences related to the distribution of health outcomes across income groups.

 The implications of our findings extend to health policy and priority-setting, particularly in terms of addressing health inequalities and the distributional impact of health interventions. Explicitly considering how interventions affect different groups is increasingly recognized as important in health economic evaluations. In this context, distributional cost-effectiveness analysis (DCEA) has been proposed as a framework to systematically account for both the overall health benefits of interventions and their distribution across different population subgroups, such as income groups.^61-63^ DCEA allows for explicit evaluation of whether health interventions disproportionately benefit certain groups, which is particularly relevant given our finding that respondents strongly disapproved of interventions that disproportionately benefit higher-income individuals. Integrating DCEA into priority-setting processes can help policy-makers identify trade-offs between improving total population health and reducing health inequalities (ie, the equity-efficiency trade-off), and implement policies that fit societal preferences regarding healthcare allocation.

###  Insights into the Preferences Regarding Prevention and Cure

 Another key finding of our study is the strong preference for preventive health interventions over curative ones, aligning with the adage “prevention is better than cure,” which was frequently mentioned in the responses to the open-ended questions. Respondents accepted 12 241 fewer healthy life years for preventive interventions, highlighting the intrinsic value of staying healthy and preventing illness. This suggests a prevention policy to maximize utility, even if it is less effective than curative interventions. The preference for prevention is mainly driven by the respondents in the “maximizing health gains and prevention” class, the only group with a significant positive marginal utility. The preference for prevention was also observed among individuals from other classes but less dominant and widespread, as also indicated by the statistically insignificant preferences. Some respondents favored curative interventions, citing the right to treatment. Notably, the type of health intervention was the least important attribute in decision-making. These findings contribute to mixed knowledge on valuing prevention versus cure.^34,35,37,39^ The research design may have influenced results, as direct comparisons favor prevention, while indirect comparisons, eg, by conducting two separate DCEs, might value curative interventions more.^36^ In addition, we did not specify the type of preventive measure in the study. It is possible that respondents’ preferences are depending on, for example, the degree of encouragement or discouragement, and the level of intrusiveness associated with different preventive measures.^38,64,65^ In the choice tasks, we indicated the effectiveness and price of the preventive intervention. An inherent assumption with this approach is thus that such an effective and feasible preventive intervention exists for the given price.

###  Insights Into the Willingness to Pay per QALY

 Respondents reported a WTP of around €41 000 per QALY, aligning with the reference value for cost-effectiveness considerations^5,6^ and comparable to other Dutch studies.^66,67^ This strengthened the evidence base on society’s WTP per QALY. The use of cost-effectiveness as a criterion for reimbursement decisions aims to achieve the best value for money, a perspective likely shared by the “maximizing health gains and prevention” class who focused on achieving maximum health benefits, and “cost-conscious” class who focused on minimizing spending. Although the attribute levels were chosen to cover a broad range surrounding the reference value, and therefore potentially nudging respondents towards this value, the possible WTP within this DCE ranged from €0 to €175 728 per QALY. Additionally, our questionnaire focused exclusively on gains in healthy life years, rather than quality-of-life improvements without lifespan extension. The public may assign greater value to improvements in quality of life than to extensions in length of life, as shown by studies from the Netherlands and internationally.^68,69^

###  Strengths and Limitations

 Understanding citizens’ preferences can be of great benefit for policy-makers in designing broadly supported health allocation policies. Our study elucidated the preferences of Dutch citizens regarding the cost, type, total health gains and distribution of health gains across income groups of health interventions. This provides valuable insights into the WTP or WTT total health gains for different distribution of health gains across income groups.

 A DCE relies on stated preferences, which are generally considered less reliable than revealed preferences. However, since the options in question are not available in real-life healthcare settings, a DCE can provide robust results when properly designed and conducted.^70^ The structured nature of questionnaire questions and the predefined attributes may have constrained respondents, potentially limiting their ability to fully express their views. This format may not have captured the nuanced perspectives of all participants, particularly those who might have more complex or non-conforming opinions. Consequently, the data collected may not entirely reflect the diversity of thought within the population. In-depth interviews may supplement DCEs with other types of information, collectively yielding a richer and more comprehensive understanding.

 In designing, formulating, and distributing the questionnaire, we aimed to enhance accessibility and inclusivity. To ensure clarity and simplicity, we selected only four attributes and composed the questionnaire at a Dutch B1 language proficiency level. Additionally, the LISS-panel organization provided digital resources and a helpdesk to facilitate access to the digital questionnaire. Despite these efforts, the subject matter remained inherently complex and abstract. Only 57% of the respondents found the choices they had to make clear, and one respondent indicated difficulties due to a language barrier. We could not comprehensively assess whether respondents fully understood the attributes, choice tasks, and their implications as intended. Respondents may have colored attributes with their own assumptions, particularly regarding the distribution of health gains between income groups. It is unclear if preferences were driven solely by income or also by other factors like health status, lifestyles, and living conditions. The introduction highlighted income-related health differences, which may have introduced bias in participants’ responses. This bias is, however, expected to be minimal as a recent study demonstrated no difference between aversion to income-related and income-caused health inequality.^27^ The representativeness might be improved by also making the questionnaire available in other languages. To mitigate potential bias from prior experience, we intentionally refrained from labeling health interventions with frequently discussed care such as physiotherapy or dental care, which increased the abstractness of the questionnaire. However, the use of abstract scenarios has been shown to result in an overestimation of the public’s WTT overall population health gains for reductions in health outcome inequalities, compared to responses elicited from real-world scenarios such as the COVID-19 pandemic.^71^ The complexity of the questionnaire and subject may have impacted its reliability and representativeness.

 The questionnaire was randomly distributed to a nationally representative panel, but the study sample was statistically significantly older and higher educated than the Dutch average. Younger and less educated individuals were less likely to participate, indicating a selection bias. Additionally, few respondents reported difficulties making ends meet or (very) bad health. However, the bias is likely minimal since background characteristics showed only occasional and inconsistent associations with preferences.

 We included four attributes of health interventions which were all considered important by respondents for decision-making. Responses to open-ended questions showed that participants weighed and traded off these attributes, confirming their significance. Furthermore, preferences spanned both ends of the attribute spectra, indicating the relevance and appropriateness of the chosen attributes and levels. However, additional factors like side effects, specific interventions, diseases treated or prevented, and patient characteristics may also influence decision-making in real-life settings but were not taken into account in this study. We did not include interaction terms between attributes or with background characteristics to keep the model interpretable, avoid overfitting and limit model complexity. Although these terms could enhance understanding of the dynamics, their inclusion requires clear hypotheses and extensive analysis. Future research, with larger samples, could address these follow-up questions.

## Conclusions

 While preferences for health gain maximization were found to be of high relevance in this study, our results also highlighted the importance of considering additional factors in healthcare reimbursement decisions, such as the distribution of health gains across the population and individual affordability. The majority favored distributing health benefits equally across all income groups. These public preferences, as elicited through this DCE, may provide valuable insights into societal values concerning the distribution of health gains, contributes to the evidence base, and may be used to inform decision-making. If the government aims to align policy-making with general societal preferences, policies should prioritize preventive interventions over equally or more effective curative interventions, and interventions that render equal gains over the population.

## Acknowledgements

 We thank all respondents for their time and sharing their opinions. We thank Tessa Jansen for her assistance in conducting the face-to-face interviews for the pre-test.

## Disclosure of artificial intelligence (AI) use

 During the preparation of this work the author(s) used “ChatRIVM”, a local and offline spin-off of OpenAI GPT, in order to improve the readability and language of the manuscript. After using this tool/service, the author(s) reviewed and edited the content as needed and take(s) full responsibility for the content of the published article.

## Ethical issues

 The research project has been assessed by the Centre for Clinical Expertise (CCE) at the National Institute for Public Health and the Environment (RIVM) in the Netherlands. The CCE concluded that the research project is exempted from further review by a medical ethics committee as it does not fulfil the specific conditions as stated in the Dutch Medical Research Involving Human Subjects Act.

## Conflicts of interest

 Authors declare that they have no conflicts of interest.

## Consent to participate

 All respondents provided informed consent to take part in the Longitudinal Internet Studies for the Social Sciences.

## Data availability statement

 The datasets used in the current study are open-access and available upon request on the Longitudinal Internet Studies for the Social Sciences website: https://www.lissdata.nl/.

## Supplementary files


Supplementary file 1. Full Questionnaire.

Supplementary file 2. Attribute Selection.

Supplementary file 3. MNL Models Stratified by Pilot and Main Study.

Supplementary file 4. Evaluation Questions and Qualitative Analysis.

Supplementary file 5. Latent Class Models.
