# Supplementary material for: What Value Do Dutch Citizens Place on Health Interventions That Provide Greater Health Gains to Lower-Income Groups? A Discrete Choice Experiment
Source: Int J Health Policy Manag. 2026 Feb 15;15:9095. doi: 10.34172/ijhpm.9095 (PMC13034220; doi:10.34172/ijhpm.9095)
Supplement: Supplementary file 1 — Full Questionnaire. [file ijhpm-15-9095-s001.pdf]

Article title: What Value Do Dutch Citizens Place on Health Interventions That Provide Greater Health Gains to Lower Income Groups? A Discrete Choice Experiment

Journal name: International Journal of Health Policy and Management (IJHPM)

Authors' information: Iris Meulman[1,2]*, Adrienne Rotteveel[1], Ellen Uiters[3], Mariëlle Cloin[2], Johan Polder[1,2], Niek Stadhouders[4,5]

[1]Center for Public Health, Healthcare and Society, National Institute for Public Health and the Environment, Bilthoven, The Netherlands.

[2]Tranzo, Tilburg School of Social and Behavioral Sciences, Tilburg University, Tilburg, The Netherlands.

[3]Netherlands School of Public & Occupational Health, Utrecht, The Netherlands.

[4]Scientific Center for Quality of Healthcare, Radboud University Medical Center, Nijmegen, The Netherlands.

[5]Department of Health Economics, School of Business and Economics & Talma Institute, Vrije Universiteit, Amsterdam, The Netherlands.

***Correspondence to:** Iris Meulman; Email: iris.meulman@rivm.nl

Citation: Meulman I, Rotteveel A, Uiters E, Cloin M, Polder J, Stadhouders N. What value do Dutch citizens place on health interventions that provide greater health gains to lower income groups? A discrete choice experiment. Int J Health Policy Manag. 2025;14:9095. doi:10.34172/ijhpm.9095

Supplementary file 1. Full Questionnaire

(Translated from Dutch to English)

Page 1

This questionnaire is about the reimbursement of interventions through basic health insurance and about differences between people with a low and a high income.

People living in the Netherlands, in general, have good health. However, there are major differences. People with a low income experience more health problems. People with incomes in the lowest 20% live an average of 51 years in good health. People with income in the highest 20% live an average of 73 years in good health. That is a difference of 22 years in good health. There are many reasons why there are differences in health between people with different incomes. For example, this may be related to personal characteristics, living conditions, livelihood security, work situation, social network and skills and access to care.

We want to know which interventions you would like to have reimbursed. Would you choose an intervention that provides the same amount of health for every patient? Or would you choose an intervention that provides greater health gains for one group of patients than for another group of patients? Does this choice also depend on the costs of the intervention and the type of intervention?

That is why we ask you to complete this questionnaire. The questionnaire starts with 3 general questions. We will then ask you to choose between 2 interventions 12 times. Finally, we will ask you to explain your choices.

Page 2

Which situation applies to you?
Multiple answers are possible

- ☐ I am employed with paid work (as an employee, as a self-employed person or own company)
- ☐ I am retired (AOW, early retirement)
- ☐ I am unemployed/jobseeker (registered with the UWV WERKbedrijf)
- ☐ I am incapacitated for work (WAO, WAZ, WIA, Wajong)
- ☐ I receive social assistance
- ☐ I am a housewife/househusband
- ☐ I am in education/I am studying

Page 3

Have you had difficulty making ends meet with your household income in the past 12 months?

- ☐ No, not at all
- ☐ No, not at all, but I have to mind my expenses

- Yes, some difficulty
- Yes, great difficulty

Page 4

How is your health in general?

- Very bad
- Bad
- Reasonably good
- Good
- Very good

Page 5

Explanation 1/2

In the Netherlands, everyone has basic health insurance (basic benefit package). The government determines what is covered by this basic health insurance. The more covered by the basic health insurance, the more expensive the premium for the basic health insurance becomes. That is why the government makes choices about what is covered and what is not.

We will ask you to choose between two interventions (intervention A and intervention B) 12 times. We want to know which of these two interventions you would like to reimburse.

Page 6

Explanation 2/2

Each intervention has consequences for an imaginary group of 10,000 patients per year. These 10,000 patients have different incomes. The 10,000 patients are divided into two groups based on their income: one group has the highest income, and the other group has the lowest income. There is no middle group.

The two interventions are each described with 4 characteristics. The two interventions only differ on these 4 characteristics.

On the following pages we explain the 4 characteristics.

Page 7

Characteristic 1: Health gains

The "average health gain" is the number of healthy life years that someone gets on average. Every year, 10,000 patients receive the intervention. Only they get extra healthy life years. Healthy life years are the extra years of life in good perceived health.

There are 3 different options for average health gain:

On average, 1 person gets **1** extra healthy life year.<br>*This means that 10,000 patients get a total of 10,000 healthy life years per year.*	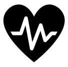
On average, 1 person gets **2** extra healthy life years.<br>*This means that 10,000 patients get a total of 20,000 healthy life years per year.*	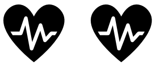
On average, 1 person gets **5** extra healthy life years.<br>*This means that 10,000 patients get a total of 50,000 healthy life years per year.*	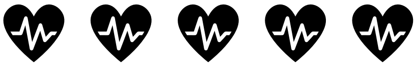

Page 8

Characteristic 2: Distribution of health gains

The "distribution of health gains between income groups" indicates how the gain in healthy life years is distributed between people with the lowest income and the highest income.

There are 3 different options for the distribution of health gains between people with the lowest and highest income:

<u>This intervention benefits people with the lowest and highest incomes</u><br><br>The health gain is divided **equally** between people with the lowest and highest incomes. This means that 50% of the health gain goes to people with the lowest income and 50% to people with the highest income.	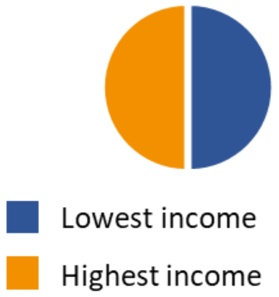
<u>This intervention mainly favors people with the highest incomes</u><br><br>People with the **highest incomes** gain **3x** as many healthy life years than people with the lowest incomes. This means that 25% of the health gain goes to people with the lowest incomes and 75% to people with the highest incomes.	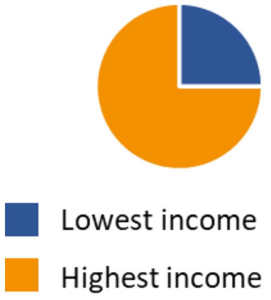
<u>This intervention mainly favors people with the lowest incomes</u><br>People with the **lowest incomes** gain **3x** as many healthy life years than people with the highest incomes. This means that 75% of the health gains go to people with the lowest incomes and 25% to people with the highest incomes.	

	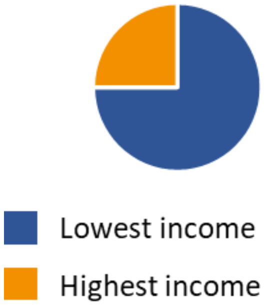

Page 9

Characteristic 3: Increase in monthly premium for all Dutch citizens

The costs for the interventions are paid from the basic health insurance. This characteristic states by how many euros per month the premium for the basic health insurance will increase if the intervention is reimbursed. These costs apply to all adult Dutch citizens. The premium will increase by this amount for the 10,000 patients and the rest of the Dutch citizens.

There are 4 different options for increases in monthly premium for the health insurance:

€0 per month	
€1 per month	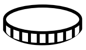
€5 per month	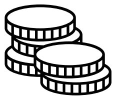
€10 per month	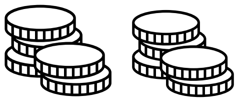

Page 10

Characteristic 4: Type of intervention

There are two options for type of intervention: interventions to make sick people (slightly) better and interventions to prevent diseases (prevention).

Intervention to make sick people (slightly) better.<br>*These types of interventions are for people who are sick. Examples of such types of interventions are: surgery and medicines.*	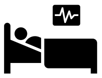
Intervention to prevent diseases (prevention)<br>*These types of interventions prevent people from getting sick. Or they prevent people from getting sicker.*	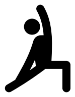

Examples of such types of interventions are: helping people to live healthier, detecting diseases early or vaccinations.	

Page 11

We will now start with the 12 choices. We ask you to advise the government which intervention should be reimbursed. First, we ask you to choose one of the two interventions (intervention A or intervention B). You have to make a choice to be able to continue. Do you neither intervention should be reimbursed? Then choose the intervention that you find the least bad. Then we ask whether you would actually advise the government to reimburse the intervention that you have chosen. If you choose 'no', that means that you would advise neither intervention.

This questionnaire is about your opinion. Therefore, there are no right or wrong answers.

Page 12 – 23

[Choice tasks with the following design for the main study]

Choice task	Option A				Option B			
	**Benefits**	**Income distribution**	**Cost**	**Type**	**Benefits**	**Income distribution**	**Cost**	**Type**
1	5	Greater health gains for lower income groups	10	Preventive	2	Health gains equally distributed	0	Curative
2	5	Health gains equally distributed	5	Preventive	2	Greater health gains for higher income groups	1	Curative
3	1	Greater health gains for lower income groups	10	Curative	5	Greater health gains for higher income groups	1	Preventive
4	1	Health gains equally distributed	5	Curative	5	Greater health gains for higher income groups	10	Curative
5	1	Greater health gains for higher income groups	0	Preventive	5	Greater health gains for higher income groups	5	Curative
6	2	Health gains equally distributed	10	Preventive	1	Greater health gains for lower income groups	0	Curative
7	1	Health gains equally distributed	1	Curative	2	Greater health gains for lower income groups	0	Preventive
8	5	Greater health gains for lower income groups	0	Curative	1	Health gains equally distributed	5	Preventive
9	2	Greater health gains for lower income groups	1	Preventive	5	Health gains equally distributed	0	Curative
10	1	Greater health gains for higher income groups	5	Preventive	2	Health gains equally distributed	10	Curative
11	1	Health gains equally distributed	0	Preventive	2	Greater health gains for lower income groups	5	Curative
12	1	Greater health gains for lower income groups	1	Curative	2	Greater health gains for higher income groups	0	Curative

Example:
Which of these two interventions do you like best?

	Treatment A	Treatment B
**Annual average health gains among 10.000 patients**	On average, 1 person gains 1 extra healthy life year 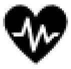	On average, 1 person gains 2 extra healthy life years 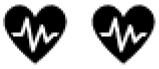
**Distribution of health gains**	This treatment benefits people with the **lowest and highest incomes.**<br><br>*The health gains are divided **equally** between people with the highest and lowest incomes*<br>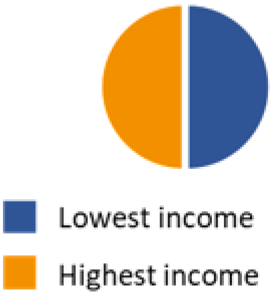	This treatment mainly benefits people with the **lowest income**<br><br>The people with the lowest income gain **3x** as many healthy life years as the people with the highest income<br>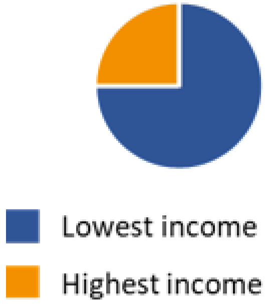
**Monthly increase in health insurance premium for all Dutch**	€5 per month 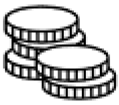	€1 per month 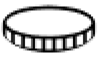
**Type of treatment**	Treatment to make sick people (slightly) better 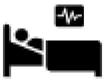	Treatment to prevent illness 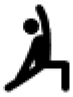
**I choose:**	☐	☐

Would you advise this treatment also in real life?

- Yes, I would advise the government to actually reimburse this treatment
- No, I would advise the government not to reimburse this treatment

Page 24

You have just made 12 choices. We would like to ask you some questions about the choices you have made.

Which characteristic did you consider most important when choosing between the two interventions? And which characteristic did you consider least important? Please, give all characteristics a number between 1 (most important) and 4 (least important). You can only use each number only once.

- ☐ Increase in health insurance premium
- ☐ Type of intervention
- ☐ Average health gain
- ☐ Distribution of health gain between income groups

Page 25

Why do you consider [insert highest ranked attribute from page 25] most important?

Page 26

Are there any other considerations that you took into account when choosing between the interventions?

Page 27

In this questionnaire you have made a choice between interventions 12 times. We are curious about what you think of these choice tasks.

**Statement**	**Totally disagree**	**Disagree**	**Not disagree, not agree**	**Agree**	**Totally agree**
I found the choices I had to make clear.	○	○	○	○	○
I missed relevant information to make choices.	○	○	○	○	○
I found the choices I had to make easy.	○	○	○	○	○
I thought it was an important subject to give my opinion on.	○	○	○	○	○
I found this research trustworthy.	○	○	○	○	○
I thought that this research steered my choices in a certain direction.	○	○	○	○	○
I was always sure about the best choice.	○	○	○	○	○

Page 28

In this questionnaire you have made a choice between interventions 12 times. We are curious about what you think of these choice tasks.

**Statement**	**Totally disagree**	**Disagree**	**Not disagree, not agree**	**Agree**	**Totally agree**
I thought this was a good method to give advice to the government as a citizen.	○	○	○	○	○
I found all the information easy to read.	○	○	○	○	○
I found the choices I had to make unpleasant.	○	○	○	○	○
I was already familiar with the subject before I participated in this research.	○	○	○	○	○
I found the choices I had to make interesting.	○	○	○	○	○
I could focus well on the choices I had to make.	○	○	○	○	○
I have carefully read and included all information in my choices.	○	○	○	○	○

Page 29

NB: Please complete the questionnaire until you return to the home screen. Only then the system will register the questionnaire as fully completed.

Finally. What did you think of this questionnaire:

**Statement**	**Definetly not 1**	**2**	**3**	4	**Definetly yes 5**
Did you find it difficult to answer the questions?	◯	◯	◯	◯	◯
Did you find the questions clear?	◯	◯	◯	◯	◯
Did the questionnaire make you think?	◯	◯	◯	◯	◯
Did you find the topic interesting?	◯	◯	◯	◯	◯
Did you enjoy filling in the questions?	◯	◯	◯	◯	◯

Page 30

Do you have any comments about this questionnaire?

- ☐ Yes ….
- ☐ No
