# Supplementary material for: What Value Do Dutch Citizens Place on Health Interventions That Provide Greater Health Gains to Lower-Income Groups? A Discrete Choice Experiment
Source: Int J Health Policy Manag. 2026 Feb 15;15:9095. doi: 10.34172/ijhpm.9095 (PMC13034220; doi:10.34172/ijhpm.9095)
Supplement: Supplementary file 2 — Attribute Selection. [file ijhpm-15-9095-s002.pdf]

Article title: What Value Do Dutch Citizens Place on Health Interventions That Provide Greater Health Gains to Lower Income Groups? A Discrete Choice Experiment

Journal name: International Journal of Health Policy and Management (IJHPM)

Authors' information: Iris Meulman[1,2]*, Adrienne Rotteveel[1], Ellen Uiters[3], Mariëlle Cloin[2], Johan Polder[1,2], Niek Stadhouders[4,5]

[1]Center for Public Health, Healthcare and Society, National Institute for Public Health and the Environment, Bilthoven, The Netherlands.

[2]Tranzo, Tilburg School of Social and Behavioral Sciences, Tilburg University, Tilburg, The Netherlands.

[3]Netherlands School of Public & Occupational Health, Utrecht, The Netherlands.

[4]Scientific Center for Quality of Healthcare, Radboud University Medical Center, Nijmegen, The Netherlands.

[5]Department of Health Economics, School of Business and Economics & Talma Institute, Vrije Universiteit, Amsterdam, The Netherlands.

***Correspondence to:** Iris Meulman; Email: iris.meulman@rivm.nl

Citation: Meulman I, Rotteveel A, Uiters E, Cloin M, Polder J, Stadhouders N. What value do Dutch citizens place on health interventions that provide greater health gains to lower income groups? A discrete choice experiment. Int J Health Policy Manag. 2025;14:9095. doi:10.34172/ijhpm.9095

Supplementary file 2. Attribute Selection

List of data sources

15 articles were identified through a literature search and informed the longlist of potential attributes.

1. Luyten J, Kessels R, Goos P, Beutels P. Public Preferences for Prioritizing Preventive and Curative Health Care Interventions: A Discrete Choice Experiment. *Value in Health.* 2015;18(2):224–233. doi:https://doi.org/10.1016/j.jval.2014.12.007
2. Ratcliffe J, Bekker HL, Dolan P, Edlin R. Examining the attitudes and preferences of health care decision-makers in relation to access, equity and cost-effectiveness: A discrete choice experiment. *Health Policy.* 2009;90(1):45–57. doi:https://doi.org/10.1016/j.healthpol.2008.09.001
3. Ward T, Mujica-Mota RE, Spencer AE, Medina-Lara A. Incorporating Equity Concerns in Cost-Effectiveness Analyses: A Systematic Literature Review. *PharmacoEconomics.* 2022;40(1):45–64. doi:10.1007/s40273-021-01094-7
4. Mirelman A, Mentzakis E, Kinter E, et al. Decision-Making Criteria among National Policymakers in Five Countries: A Discrete Choice Experiment Eliciting Relative Preferences for Equity and Efficiency. *Value in Health.* 2012;15(3):534–539. doi:https://doi.org/10.1016/j.jval.2012.04.001
5. O'Neill J, Tabish H, Welch V, et al. Applying an equity lens to interventions: using PROGRESS ensures consideration of socially stratifying factors to illuminate inequities in health. *Journal of Clinical Epidemiology.* 2014;67(1):56–64. doi:https://doi.org/10.1016/j.jclinepi.2013.08.005
6. Vallgårda S. Social inequality in health: Dichotomy or gradient?: A comparative study of problematizations in national public health programmes. *Health Policy.* 2008;85(1):71–82. doi:https://doi.org/10.1016/j.healthpol.2007.07.004
7. Baji P, García-Goñi M, Gulácsi L, Mentzakis E, Paolucci F. Comparative analysis of decision maker preferences for equity/efficiency attributes in reimbursement decisions in three European countries. *The European Journal of Health Economics.* 2016;17(7):791–799. doi:10.1007/s10198-015-0721-x
8. Jehu-Appiah C, Baltussen R, Acquah C, et al. Balancing Equity and Efficiency in Health Priorities in Ghana: The Use of Multicriteria Decision Analysis. *Value in Health.* 2008;11(7):1081–1087. doi:https://doi.org/10.1111/j.1524-4733.2008.00392.x
9. Paolucci F, Mentzakis E, Defechereux T, Niessen LW. Equity and efficiency preferences of health policy makers in China—a stated preference analysis. *Health Policy and Planning.* 2015;30(8):1059–1066. doi:10.1093/heapol/czu123
10. Franken M, Koolman X. Health system goals: A discrete choice experiment to obtain societal valuations. *Health Policy.* 2013;112(1):28–34. doi:https://doi.org/10.1016/j.healthpol.2012.12.013
11. Cadham CJ, Prosser LA. Eliciting Trade-Offs Between Equity and Efficiency: A Methodological Scoping Review. *Value in Health.* 2023;26(6):943–952. doi:https://doi.org/10.1016/j.jval.2023.02.006
12. van de Wetering EJ, van Exel NJA, Rose JM, Hoefman RJ, Brouwer WBF. Are some QALYs more equal than others? *The European Journal of Health Economics.* 2016;17(2):117–127. doi:10.1007/s10198-014-0657-6
13. Tsuchiya A, Dolan P. Do NHS clinicians and members of the public share the same views about reducing inequalities in health? *Social Science & Medicine.* 2007;64(12):2499–2503. doi:https://doi.org/10.1016/j.socscimed.2007.03.013
14. Gu Y, Lancsar E, Ghijben P, Butler JRG, Donaldson C. Attributes and weights in health care priority setting: A systematic review of what counts and to what extent. *Social Science & Medicine.* 2015;146:41–52. doi:https://doi.org/10.1016/j.socscimed.2015.10.005

15. Mortimer D, Segal L. Is the value of a life or life-year saved context specific? Further evidence from a discrete choice experiment. *Cost Eff Resour Alloc.* 2008;6:8. doi:10.1186/1478-7547-6-8

Long list of potential attributes

Table S 1. Long list of potential attributes

Category	Attributes	Levels	Source
(In)equality	Deployment of resources / Target group	• Universal<br>• Proportional to SES<br>• Proportional to health/disease severity<br>• Proportional to lifestyle<br>• Proportional to age	1, 4, 5, 7, 8, 9, 12, 14, 15
(In)equality	Benefits / health benefits (social)<br>*In terms of QALYs, healthy life expectancy, ...*	• Proportional<br>• Benefits vary according to SES<br>• Benefits vary according to health/disease severity (sometimes need is seen as a separate category)<br>• Benefits vary by lifestyle (fully, partial, non)<br>• Benefits vary by age	2, 5, 7, 14, 15
(In)equality	Cause of inequalities	• (Health) behaviour<br>• Social relationships<br>• Underlying social systems (structural) / unequal opportunities	6
(In)equality	Deployment of resources	• welfare state model<br>• Targeting vulnerable groups<br>• Universal	6, 11
(In)equality	Behavioral/lifestyle-related condition	• Fully<br>• Partial<br>• Not at all	1, 12, 15
(In)equality	Degree of contribution to society	• Caring for dependents<br>• Does not care for dependents, or<br>• Working<br>• Pensioner	12
(In)equality	Type of disorder	• Chronic<br>• Acute	1
(In)equality	Severity of the disease	• Light<br>• Serious	4, 7, 8, 9
Effectiveness	Size	• Large group of people<br>• Small group of people	4, 7, 8, 9, 14
Effectiveness	Individual health gains	• Little health gain (QALY, DALY, LY etc)<br>• Lots of health gains (QALY, DALY, LYs etc)	2, 4, 7, 9, 12, 14

Effectiveness	Efficiency vs. equity	• Smaller inequalities with less total health gain<br>• Greater inequality with more total health gains<br>• Efficiency indicators in terms of total health gains for the population, health gains for an individual and cost-effectiveness	3, 11, 13, 14
Effectiveness	Aspects/conditions of effectiveness	• Accessibility<br>• Acceptability<br>• Effective in vulnerable groups<br>• Knowledge/information gap and required language and health skills<br>• Connecting with (health) beliefs and trust	5
Effectiveness	Probability of effective intervention	• X%<br>• X%<br>• X%	1, 14
Costs/ Financing	Monthly increase in health insurance premium	• €X<br>• €X<br>• €X	14
Costs/financing	Cost	• Low<br>• Medium<br>• High	14, 15
Costs/ Financing	Cost-effectiveness	• Cost effective<br>• Not cost-effective	4, 7, 8, 9, 14
Costs/ Financing	Willingness to subsidize	• Less than X% is paid from public funds<br>• More than X% is paid from public funds	7, 9
Costs/financing	Personal contribution (how much does the patient have to contribute)	• Nothing<br>• Quarter<br>• Half<br>• Everything	15
Initial situation	Initial utility	• X utility<br>• X utility<br>• X utility	12
Initial situation	Initial life expectancy	• X years<br>• X years<br>• X years	10
Other	Type / purpose of the measure	• Curative<br>• Preventive	1, 14, 15
Other	Time aspect	• Short-term costs<br>• Long-term benefits	
Other	Quality vs. quantity	• Life-prolonging treatment<br>• Quality-improving treatment	14

These attributes in the longlist were ranked on relevance to the research question, distinctiveness, and simplicity reduced this to 14 attributes. Finally, for each characteristic of the health interventions mentioned in the research question, we decided to chose one attribute based on its relevance and synergistic compatibility from the table underneath. The final chosen attribute is marked grey.

Table S 2. Remaining attributes sorted in categories

**Cost**	**Distribution to SES and other relevant characteristics**	**Health effects**	**Type of intervention**
Increase in health insurance premium for a certain period	Difference in health benefits by health status	Scope of the measure (part of the population/ number of people/ number of patients)	Prevention vs. cure
Increase tax for a certain period	Difference in health benefits by SES	QALY gain total at population level	
Total costs (for the government?)	Difference in health benefits by lifestyle	QALY gain per persoon	
Collective vs. Individual financing	Difference in health benefits by age		
Personal contribution patient	Difference in health benefits by productivity (extent to which someone can contribute to society)		
	Difference in health benefits by severity of disease		
