# Supplementary material for: What Value Do Dutch Citizens Place on Health Interventions That Provide Greater Health Gains to Lower-Income Groups? A Discrete Choice Experiment
Source: Int J Health Policy Manag. 2026 Feb 15;15:9095. doi: 10.34172/ijhpm.9095 (PMC13034220; doi:10.34172/ijhpm.9095)
Supplement: Supplementary file 3 — MNL Models Stratified by Pilot and Main Study. [file ijhpm-15-9095-s003.pdf]

Article title: What Value Do Dutch Citizens Place on Health Interventions That Provide Greater Health Gains to Lower Income Groups? A Discrete Choice Experiment

Journal name: International Journal of Health Policy and Management (IJHPM)

Authors' information: Iris Meulman[1,2]*, Adrienne Rotteveel[1], Ellen Uiters[3], Mariëlle Cloin[2], Johan Polder[1,2], Niek Stadhouders[4,5]

[1]Center for Public Health, Healthcare and Society, National Institute for Public Health and the Environment, Bilthoven, The Netherlands.

[2]Tranzo, Tilburg School of Social and Behavioral Sciences, Tilburg University, Tilburg, The Netherlands.

[3]Netherlands School of Public & Occupational Health, Utrecht, The Netherlands.

[4]Scientific Center for Quality of Healthcare, Radboud University Medical Center, Nijmegen, The Netherlands.

[5]Department of Health Economics, School of Business and Economics & Talma Institute, Vrije Universiteit, Amsterdam, The Netherlands.

***Correspondence to:** Iris Meulman; Email: iris.meulman@rivm.nl

Citation: Meulman I, Rotteveel A, Uiters E, Cloin M, Polder J, Stadhouders N. What value do Dutch citizens place on health interventions that provide greater health gains to lower income groups? A discrete choice experiment. Int J Health Policy Manag. 2025;14:9095. doi:10.34172/ijhpm.9095

Supplementary file 3. MNL Models Stratified by Pilot and Main Study

Table S 1. Multinomial logit models stratified by pilot and main study

	Pilot study<br>N = 136<br>*β-coefficient*	Main study<br>N = 478<br>*β-coefficient*	Combined<br>N = 614<br>*β-coefficient*
**Attributes**			
Health benefits (10,000 healthy life years)	0.253 (0.207-0.298)	0.218 (0.194-0.243)	0.221 (0.198-0.243)
Equally distributed health gains (50/50)	Ref	Ref	Ref
Greater health gains for higher income groups (75/25)	-1.651 (-1.893--1.409)	-1.399 (-1.527--1.271)	-1.427 (-1.547--1.307)
Greater health gains for lower income groups (25/75)	-0.374 (-0.551--0.197)	-0.308(-0.397 --0.218)	-0.315 (-0.395--0.235)
Increase of health insurance premium (€, yearly)	-0.008(-0.010--0.006)	-0.008 (-0.009--0.007)	-0.008 (-0.009--0.007)
Curative health intervention	Ref	Ref	Ref
Preventive health intervention	0.277 (0.114-0.439)	0.275 (0.203-0.347)	0.270 (0.204-0.336)
ASC (alternative B)	0.106 (-0.016-0.229)	0.021 (-0.029-0.071)	0.041 (-0.006-0.088)
ASC (opt-out)	-0.699 (-0.952--0.446)	-0.558 (-0.699--0.418)	-0.572 (-0.695--0.449)
Scaling parameter opt-out model	1.035 (0.959-1.111)	1.126 (1.072-1.180)	1.105 (1.059-1.151)
Scaling parameter pilot	-	-	1.097 (0.954-1.241)

ASC = alternative specific constant
