# Supplementary material for: What Value Do Dutch Citizens Place on Health Interventions That Provide Greater Health Gains to Lower-Income Groups? A Discrete Choice Experiment
Source: Int J Health Policy Manag. 2026 Feb 15;15:9095. doi: 10.34172/ijhpm.9095 (PMC13034220; doi:10.34172/ijhpm.9095)
Supplement: Supplementary file 4 — Evaluation Questions and Qualitative Analysis. [file ijhpm-15-9095-s004.pdf]

Article title: What Value Do Dutch Citizens Place on Health Interventions That Provide Greater Health Gains to Lower Income Groups? A Discrete Choice Experiment

Journal name: International Journal of Health Policy and Management (IJHPM)

Authors' information: Iris Meulman[1,2]*, Adrienne Rotteveel[1], Ellen Uiters[3], Mariëlle Cloin[2], Johan Polder[1,2], Niek Stadhouders[4,5]

[1]Center for Public Health, Healthcare and Society, National Institute for Public Health and the Environment, Bilthoven, The Netherlands.

[2]Tranzo, Tilburg School of Social and Behavioral Sciences, Tilburg University, Tilburg, The Netherlands.

[3]Netherlands School of Public & Occupational Health, Utrecht, The Netherlands.

[4]Scientific Center for Quality of Healthcare, Radboud University Medical Center, Nijmegen, The Netherlands.

[5]Department of Health Economics, School of Business and Economics & Talma Institute, Vrije Universiteit, Amsterdam, The Netherlands.

***Correspondence to:** Iris Meulman; Email: iris.meulman@rivm.nl

Citation: Meulman I, Rotteveel A, Uiters E, Cloin M, Polder J, Stadhouders N. What value do Dutch citizens place on health interventions that provide greater health gains to lower income groups? A discrete choice experiment. Int J Health Policy Manag. 2025;14:9095. doi:10.34172/ijhpm.9095

Supplementary file 4. Evaluation Questions and Qualitative Analysis

Additional information on the evaluation questions

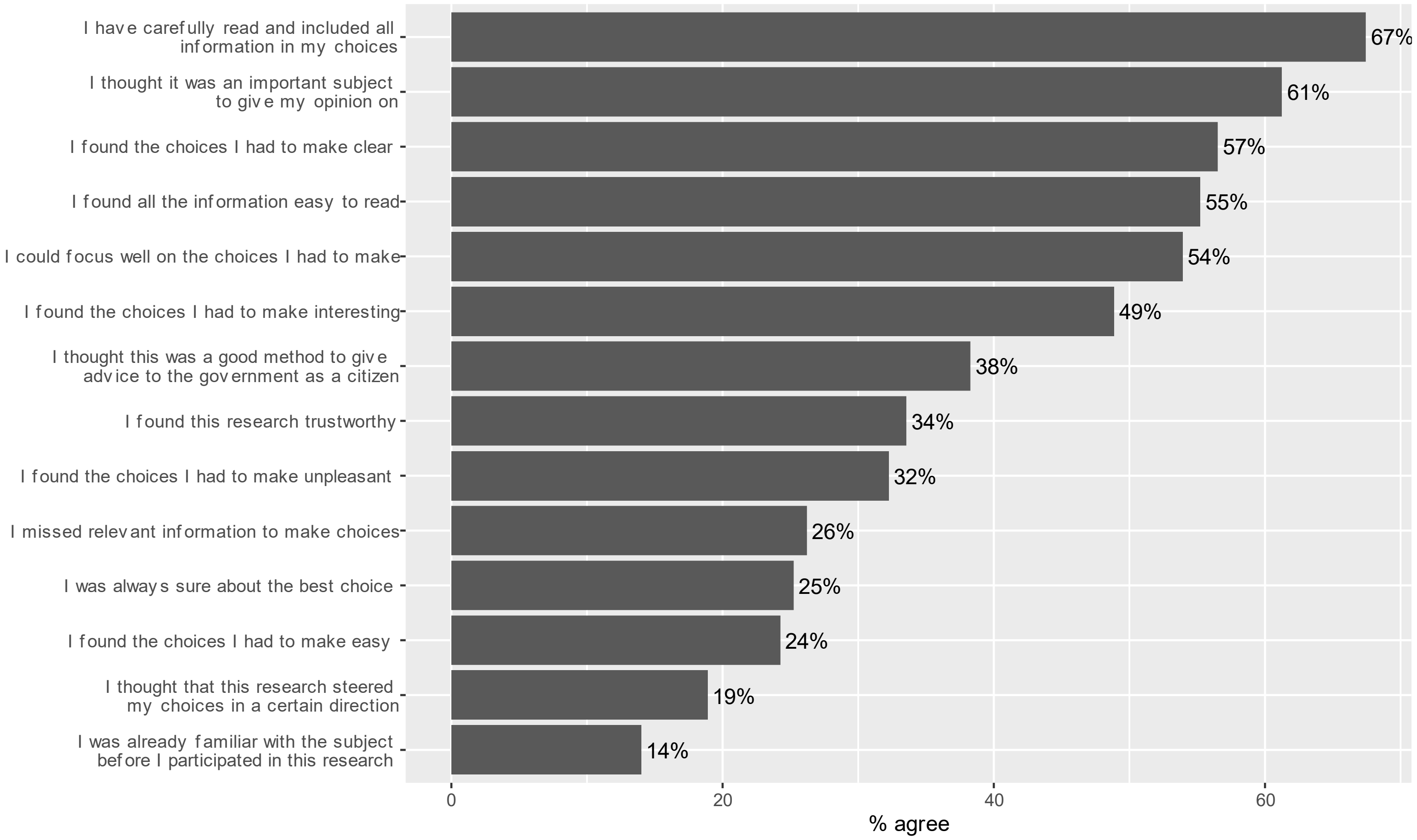

Figure S 1. Scores of evaluation statements

Coding tree used for qualitative analysis

Table S 1. Coding tree used for qualitative analysis

**Attribute**	**Argument (in alphabetical order)**	**Sub-argument (if applicable; in alphabetical order)**
Annual average gain in healthy life years	Added value for society	
	Cost-effectiveness	
	Costs	
	Everyone benefits	
	Goes without saying	
	Health is important	
	Individual benefit	
	Most effective	
	Large differences between alternatives	
	Length of life	
	Long term	
	Quality of life	
	Ultimate goal	
Distribution of total health gains by income level	Against more health gains for higher income groups	
	Equality	Equal chances
		Equal outcomes
		Equal treatment/right
	Pro more health gains for lower income groups	Decreasing health differences
		Higher income groups have more resources
		More support for lower income groups
Increase in monthly healthcare premium	Affects every Dutch citizen	
	Affordability premium	
	Cost-effectiveness	
	Differential impact low vs high income groups	

	Displacement	
	Small amount	
Type of health intervention	Cure over prevention	Important that everyone can be treated
		Prevention is ineffective
		Prevention is people's own responsibility
	Prevention over cure	Externalities of prevention
		Long-term cost-savings of prevention
		Preventing diseases/treatments
		Prevention is better than cure
Other considerations taken into account	Accessibility	
	Appropriate care	
	Characteristics patient	
	Fairness	
	Side-effects	
	Type of treatment	
	Waste of money/issues healthcare system	

Findings of qualitative analysis

The text below is translated from Dutch to English with DeepL.com (free version). The translations by DeepL were critically checked and, if necessary, adjusted by the authors.

General findings with regards to the four attributes

Annual average gain in healthy life years

People report a variety of reasons to explain why they consider gains in healthy life years to be most important attribute. A frequently mentioned reason is that health is what it's all about, that it is the goal of healthcare. So that should be the focus. Other reasons mentioned include that everyone benefits from health gains, that living a long(er) healthy life is very important,

that health is the most important thing, and that society benefits when people have more healthy years of life (during which they can be productive and contribute to society).
Some people mainly justify why they opted for interventions with significant health gains. They indicate that significant health gains indicate that the intervention is effective or yields significant results, and that they consider this important.
Finally, many people mention that the investment made must yield sufficient results. They therefore weigh the health gains against the costs.

Distribution of total health gains by income level

The qualitative responses reveal that people have different opinions about the distribution of health gains.
A group of respondents believe it is important for people with lower incomes to benefit more from health gains. Some of them believe it is important that more health gains are distributed to people with lower incomes because people with higher incomes can pay for treatment and prevention themselves, while people with lower incomes are dependent on what is paid for by the government. They believe it is important to give people with lower incomes a chance too. Others mainly want to do something about the existing health inequalities, sometimes because they were shocked by the information about health inequalities at the beginning of the questionnaire.
Another group of respondents indicated that equality is important to them and that income should not be a factor. Some even wrote that they find it reprehensible or unfair to discriminate on the basis of income when it comes to health. However, these people are not always clear about whether the importance they attach to equality means that health benefits should be distributed equally or that it is important for everyone to have equal health. Some people, however, describe more clearly what they mean by equality. Three groups can be distinguished here: 1) people who attach importance to equal rights to care, 2) people who attach importance to equal opportunities, and 3) people who attach importance to equal health gains. People in the first group state that everyone has the right to be treated the same in healthcare. Everyone has the right to receive the same, good care, regardless of income. People in the second group believe that everyone deserves equal opportunities for health. This should not depend on a person's income. People in the third group believe that there should be no difference in health gains based on a person's income.
Finally, a few respondents mention that they are primarily opposed to greater health gains for high-income earners because higher-income earners are already better off than lower-income

earners. However, they do not express a preference for equal distribution versus distribution that results in greater health gains for low-income earners.

Increase in monthly healthcare premium

The reason most frequently given by participants when asked why they consider premium to be the most important attribute is that current premiums are already very high. Some people mention that this makes premiums unaffordable for people with low incomes or for themselves. Others mention that it is particularly important for low-income groups that healthcare premiums are not increased too much.

A less frequently mentioned reason why premium is important is that an increase in premium affects every Dutch citizen. Because it affects every Dutch citizen, people believe it is important to carefully consider premium increases.

Finally, there are also people who explicitly weigh the increase in premium against the health benefits. These people have critically considered how to achieve as many health benefits as possible with as few resources as possible.

Type of health intervention

People who considered the attribute “type of health intervention” to be most important generally did so because of a strong preference for either cure or prevention.

The most frequently cited reason for preferring cure was that people consider it important that if someone becomes ill, that person can receive treatment. Some people added that they believe everyone has a right to treatment.

People who prefer prevention indicate that they consider prevention important. Some mention that they believe there is currently too little focus on prevention. A large proportion of respondents explicitly quote the expression “prevention is better than cure” as justification for the importance of prevention. Many people also explicitly link prevention to the prevention of diseases and, subsequently, treatments, and mention that more health benefits can be achieved with prevention than with cure. Some of the participants also indicate that they believe prevention will lead to cost savings in the long term. Finally, a few mention that preventing health problems in one person also leads to fewer health problems among the people surrounding that person.

General findings on other consideration taken into account

After asking which attribute people found most important, they were also asked which other considerations played a role. These were often the other attributes included the DCE. For example, a number of people mentioned that they also took costs into account in their decision, particularly in relation to the health benefits. Many people also indicated that they had considered the type of treatment, with most mentioning that they considered prevention to be important. A number of people indicated that they were opposed to prevention because they believe that prevention does not work and that prevention is the responsibility of individuals. Health gains were also mentioned as another consideration that played a role, sometimes in comparison with other criteria such as the premium and the type of treatment. Furthermore, people mentioned that the distribution of health gains had also played a role, with some favouring larger health gains for low-income groups and some favouring equality, with the same justifications as mentioned above.

A few considerations were also mentioned that people took into account, even though they were not addressed in the DCE. A number of people mentioned inefficiencies in the Dutch healthcare system that they believe need to be addressed, such as waste and misappropriation of funds. People also mentioned that they considered a fair distribution of health gains to be important. They did not specify what they considered to be a fair distribution. A few people also mentioned side effect
