# Supplementary material for: What Value Do Dutch Citizens Place on Health Interventions That Provide Greater Health Gains to Lower-Income Groups? A Discrete Choice Experiment
Source: Int J Health Policy Manag. 2026 Feb 15;15:9095. doi: 10.34172/ijhpm.9095 (PMC13034220; doi:10.34172/ijhpm.9095)
Supplement: Supplementary file 5 — Latent Class Models. [file ijhpm-15-9095-s005.pdf]

Article title: What Value Do Dutch Citizens Place on Health Interventions That Provide Greater Health Gains to Lower Income Groups? A Discrete Choice Experiment

Journal name: International Journal of Health Policy and Management (IJHPM)

Authors' information: Iris Meulman[1,2]*, Adrienne Rotteveel[1], Ellen Uiters[3], Mariëlle Cloin[2], Johan Polder[1,2], Niek Stadhouders[4,5]

[1]Center for Public Health, Healthcare and Society, National Institute for Public Health and the Environment, Bilthoven, The Netherlands.

[2]Tranzo, Tilburg School of Social and Behavioral Sciences, Tilburg University, Tilburg, The Netherlands.

[3]Netherlands School of Public & Occupational Health, Utrecht, The Netherlands.

[4]Scientific Center for Quality of Healthcare, Radboud University Medical Center, Nijmegen, The Netherlands.

[5]Department of Health Economics, School of Business and Economics & Talma Institute, Vrije Universiteit, Amsterdam, The Netherlands.

***Correspondence to:** Iris Meulman; Email: iris.meulman@rivm.nl

Citation: Meulman I, Rotteveel A, Uiters E, Cloin M, Polder J, Stadhouders N. What value do Dutch citizens place on health interventions that provide greater health gains to lower income groups? A discrete choice experiment. Int J Health Policy Manag. 2025;14:9095. doi:10.34172/ijhpm.9095

Supplementary file 5. Latent Class Models

Model diagnostics 1-9 latent classes

Table S 1. Log-likelihood and BIC of joint latent class choice model. BIC = Bayesian Information Criterion. MNL = multinomial logit model

Number of classes	Log-likelihood	BIC	Rho-squared	Number of parameters	Mean probability of class allocation[1]								
					1	2	3	4	5	6	7	8	9
1 class (MNL)	-11410.42	22900.99	0.1350	9	100%								
2 classes	-10921.45	22006.06	0.1714	17	48%	52%							
3 classes	-10544.15	21328.25	0.1994	25	28%	35%	37%						
4 classes	-12989.53	20851.63	0.2223	33	25%	27%	28%	20%					
5 classes	-9973.56	20340.64	0.2414	41	19%	21%	18%	26%	16%				
6 classes	-9795.68	20061.67	0.2543	49	16%	15%	16%	23%	16%	14%			
7 classes	-9656.20	19859.50	0.2642	57	21%	16%	16%	15%	12%	6%	15%		
8 classes	9533.21	19690.29	0.2730	65	11%	16%	9%	14%	15%	5%	18%	12%	
9 classes	-9454.19	19609.04	0.2783	73	12%	11%	11%	8%	16%	6%	12%	15%	10%

[1] Note that the mean probability of class allocation may not correspond with the posterior class membership probabilities of individuals

Multinomial logit model per latent class

Table S 2. Jointly estimated MNL model per latent class.

	Class 1<br>*β-coefficient (95% CI)*	Class 2<br>*β-coefficient (95% CI)*	Class 3<br>*β-coefficient (95% CI)*	Class 4<br>*β-coefficient (95% CI)*
**Attributes and covariates**				
Health benefits (10,000 healthy life years)	0.421 (0.310-0.533)	0.180 (0.107-0.253)	0.213 (0.101-0.324)	0.276 (0.161-0.392)
Distribution of health gains by income				
Equally distributed (50/50)	Ref	Ref	Ref	Ref
Greater health gains for higher income groups (75/25)	-0.720 (-1.066--0.374)	-2.191 (-2.725--1.658)	-2.699 (-3.249--2.148)	-1.079 (-1.431--0.727)
Greater health gains for lower income groups (25/75)	-0.023 (-0.280-0.235)	-1.692 (-2.197--1.187)	0.216 (-0.400-0.831)	-0.134 (-0.425-0.157)
Health insurance premium (€, yearly)	-0.004 (-0.008--0.001)	-0.007 (-0.009--0.005)	-0.008 (-0.013--0.004)	-0.027 (-0.033--0.022)
Type of health intervention				
Curative	Ref	Ref	Ref	Ref
Preventive	0.875 (0.592-1.159)	0.100 (-0.165-0.364)	0.113 (-0.202-0.428)	0.262 (-0.091-0.615)
Pilot parameter				
Latent class intercept	0	0.092 (-0.261-0.444)	0.142 (-0.232-0.516)	-0.218 (-0.591-0.155)
ASC (alternative B)	0.180 (0.001-0.359)	-0.167 (-0.332--0.002)	-0.012 (-0.147-0.123)	0.232 (0.043-0.422)

ASC (opt-out)	0.255 (-0.249-0.759)	-0.489 (-1.427-0.45)	-1.358 (-2.546--0.17)	-1.055 (-1.672--0.438)
Scaling parameter opt out model		1.069 (0.991-1.148)		
Scaling parameter pilot		1.087 (0.959-1.214)		
**Model output**				
Number of observations	149	168	177	120
Final LL per class	-13246.18	-13782.16	--13181.26	-14360.01
Final LL joint model		-10267.45		
AIC		20600.89		
BIC		20851.63		
Rho-squared		0.2198		

MNL = multinomial logit model, LCA = latent class analysis, ASC = alternative specific constant, LL = Log-likelihood, AIC = Akaike Information Criterion, BIC = Bayesian Information Criterion. Class 1: "preferring lower income groups benefitting more", Class 2: "equal distribution of health gains", Class 3: "maximizing health gains and prevention" and Class 4: "cost-conscious".

Associations between posterior class membership and background characteristics

Table S 3. Univariate associations between posterior class membership and personal background characteristics

	Class 1	Class 2	Class 3	Class 4
	OR (95% CI)	*OR (95% CI)*	*OR (95% CI)*	*OR (95% CI)*
Sex				
Men	Ref	Ref	Ref	Ref
Women	0.58 (0.40-0.85)	1.46 (1.02-2.09)	0.94 (0.66-1.33)	1.26 (0.85-1.88)
Age				
18-24 years	1.79 (0.80-4.04)	2.13 (1.01-4.49)	0.22 (0.08-0.59)	1.18 (0.45-3.06)
25-34 years	2.20 (1.25-3.90)	1.66 (0.96-2.86)	0.16 (0.07-0.33)	1.64 (0.87-3.09)
35-44 years	2.07 (1.19-3.61)	0.96 (0.54-1.71)	0.42 (0.25-0.73)	1.52 (0.82-2.82)
45-54 years	1.67 (0.89-3.14)	1.67 (0.93-2.99)	0.25 (0.12-0.50)	1.69 (0.86-3.30)
55-64 years	1.12 (0.63-1.99)	1.03 (0.61-1.75)	0.63 (0.39-1.01)	1.75 (0.98-3.09)
> 65 years	Ref	Ref	Ref	Ref
Education				
Low	0.65 (0.38-1.11)	1.30 (0.81-2.09)	1.79 (1.12-2.85)	0.51 (0.28-0.92)
Middle	0.87 (0.58-1.31)	1.11 (0.75-1.66)	1.31 (0.88-1.96)	0.74 (0.48-1.15)
High	Ref	Ref	Ref	Ref
Household monthly net income				
< €2000	Ref	Ref	Ref	Ref
€2000-3000	0.98 (0.49-1.94)	1.27 (0.68-2.39)	1.67 (0.93-3.00)	0.30 (0.14-0.67)

€3000-4000	1.18 (0.60-2.35)	0.78 (0.39-1.54)	1.31 (0.71-2.41)	0.77 (0.39-1.52)
€4000-5000	1.48 (0.72-3.02)	1.63 (0.83-3.21)	0.96 (0.49-1.88)	0.29 (0.12-0.72)
€5000-6000	1.25 (0.60-2.64)	1.03 (0.50-2.12)	0.56 (0.27-1.17)	1.36 (0.68-2.71)
> €6000	1.52 (0.76-3.05)	1.54 (0.80-3.00)	0.37 (0.17-0.79)	0.98 (0.50-1.95)
Income unknown	1.42 (0.66-3.03)	1.26 (0.60-2.62)	0.73 (0.35-1.54)	0.77 (0.35-1.67)
Difficulties making ends meet				
No difficulties	Ref	Ref	Ref	Ref
No difficulties, but minding expenses	0.96 (0.63-1.45)	0.74 (0.49-1.11)	1.71 (1.17-2.51)	0.74 (0.47-1.17)
Some/large difficulties	0.84 (0.45-1.56)	1.16 (0.66-2.02)	1.44 (0.82-2.52)	0.61 (0.30-1.24)
Employment situation				
Employed	Ref	Ref	Ref	Ref
Retired	0.54 (0.35-0.83)	0.77 (0.51-1.17)	3.35 (2.26-4.98)	0.54 (0.33-0.86)
Student	1.22 (0.58-2.54)	1.30 (0.62-2.71)	0.99 (0.42-2.38)	0.52 (0.20-1.40)
Unemployed	0.49 (0.25-0.97)	1.41 (0.82-2.44)	1.50 (0.83-2.74)	0.87 (0.46-1.63)
Self-reported health				
(Very) good	2.28 (0.66-7.86)	1.80 (0.60-5.44)	0.38 (0.16-0.90)	0.91 (0.33-2.52)
Average health	1.60 (0.45-5.70)	1.54 (0.49-4.79)	0.77 (0.31-1.88)	0.62 (0.21-1.82)
(Very) bad	Ref	Ref	Ref	Ref

Opt-out behavior per latent class

Table S 4. Frequency of choosing opt-out per latent class

Number of times opt-out	Class 1	Class 2	Class 3	Class 4
0	42.3%	14.9%	35.6%	21.7%
1	11.4%	5.4%	18.6%	15.8%
2	8.7%	6.0%	13.6%	15.0%
3	16.1%	12.5%	11.3%	13.3%
4	10.1%	12.5%	9.0%	17.5%
5	3.4%	7.7%	8.5%	5.8%
6	4.7%	10.1%	2.3%	7.5%
7	2.7%	8.3%	0.6%	1.7%
8	0.7%	7.1%	0%	1.7%
9	0%	4.2%	0%	0%
10	0%	1.8%	0.6%	0%
11	0%	4.8%	0%	0%
12	0%	4.8%	0%	0%

Qualitative findings per latent class

Class 1 - "maximizing health gains and prevention" class

People in this cluster who chose health benefits as the most important attribute cited a large variety of reasons for this. No clear trend was observed in this variety of reasons.

People in this cluster who chose the type of treatment as the most important attribute mainly indicated that prevention was very important to them. Some mentioned that prevention prevents healthcare and therefore healthcare costs. However, there was also a group that indicated that cure was important to them.

No clear pattern emerged from the answers to the question about what other considerations were important.

Class 2 - "equal distribution of health gains" class

People in this cluster who chose the distribution of health gains as the most important attribute refer strikingly often to equality (equal opportunities, equal distribution, equal treatment, equality, class equality) in their reasoning. Some also mention that a person's income should not matter when it comes to health gains/healthcare.

People in this cluster who chose health gains as the most important attribute often mention that this benefits everyone.
The term equality also comes up remarkably often in the question about what other considerations played a role. In particular, equal treatment of people with low and high incomes is often mentioned.

Class 3 – "preferring equal health gains or lower income groups benefitting more" class
People in this cluster who chose the distribution of health gains as the most important attribute cite various arguments. Part of the respondents believe that lower incomes deserve more support from the government, some of who explicitly state that they believe this because higher incomes are already better off in many respects and are better able to pay for things themselves. Another group of respondents believe that everyone has an equal right to healthcare or that everyone should have an equal opportunity to be healthy. Income should not be a factor in this.
People in this cluster who chose premium as the most important attribute mention that the premium is becoming unaffordable. Some of them mention that this is specifically a problem for lower incomes.
However, what is most striking are the answers to the open question about other considerations. A large proportion of the people in this cluster responded to that question by saying that they believe there should be no health gap between rich and poor and that everyone has an equal right to healthcare. Another large proportion mentioned the importance of prevention as a consideration they also took into account. A smaller proportion linked both considerations: they believe that lower incomes in particular benefit from prevention and therefore consider prevention important in reducing health inequalities.

Class 4 - "cost-conscious" class
People in this cluster who chose premium as the most important attribute mainly justify this by stating that the premium is already very high and that they consider it important that it does not rise further. Some mention that the premium is already unaffordable for lower incomes.
People in this cluster who chose health gains as the most important attribute give various reasons for this. Some indicate that this reflects the added value for society, while others say they looked at the greatest health gains for the lowest premium increase. A variety of other

reasons are also mentioned, such as health being the most important thing and because that is the purpose of healthcare.

No clear pattern emerged from the answers to the question of what other considerations were important.
